# Distance-dependent duplex DNA destabilization proximal to G-quadruplex/*i*-motif sequences

**DOI:** 10.1093/nar/gkt476

**Published:** 2013-06-14

**Authors:** Sebastian L. B. König, Julian L. Huppert, Roland K. O. Sigel, Amanda C. Evans

**Affiliations:** ^1^Cavendish Laboratory, University of Cambridge, JJ Thomson Avenue, Cambridge CB3 0HE, UK, ^2^Institute of Inorganic Chemistry, University of Zurich, Winterthurerstrasse 190, 8057 Zurich, Switzerland and ^3^University of Nice-Sophia Antipolis, UMR 7272 CNRS, Institut de 40 Chimie de Nice, 28 Avenue Valrose, 06108 Nice, France

## Abstract

G-quadruplexes and *i*-motifs are complementary examples of non-canonical nucleic acid substructure conformations. G-quadruplex thermodynamic stability has been extensively studied for a variety of base sequences, but the degree of duplex destabilization that adjacent quadruplex structure formation can cause has yet to be fully addressed. Stable *in vivo* formation of these alternative nucleic acid structures is likely to be highly dependent on whether sufficient spacing exists between neighbouring duplex- and quadruplex-/*i*-motif-forming regions to accommodate quadruplexes or *i-*motifs without disrupting duplex stability. Prediction of putative G-quadruplex-forming regions is likely to be assisted by further understanding of what distance (number of base pairs) is required for duplexes to remain stable as quadruplexes or *i*-motifs form. Using oligonucleotide constructs derived from precedented G-quadruplexes and *i*-motif-forming *bcl-2* P1 promoter region, initial biophysical stability studies indicate that the formation of G-quadruplex and *i*-motif conformations do destabilize proximal duplex regions. The undermining effect that quadruplex formation can have on duplex stability is mitigated with increased distance from the duplex region: a spacing of five base pairs or more is sufficient to maintain duplex stability proximal to predicted quadruplex/*i-*motif-forming regions.

## INTRODUCTION

**Figure 1.**
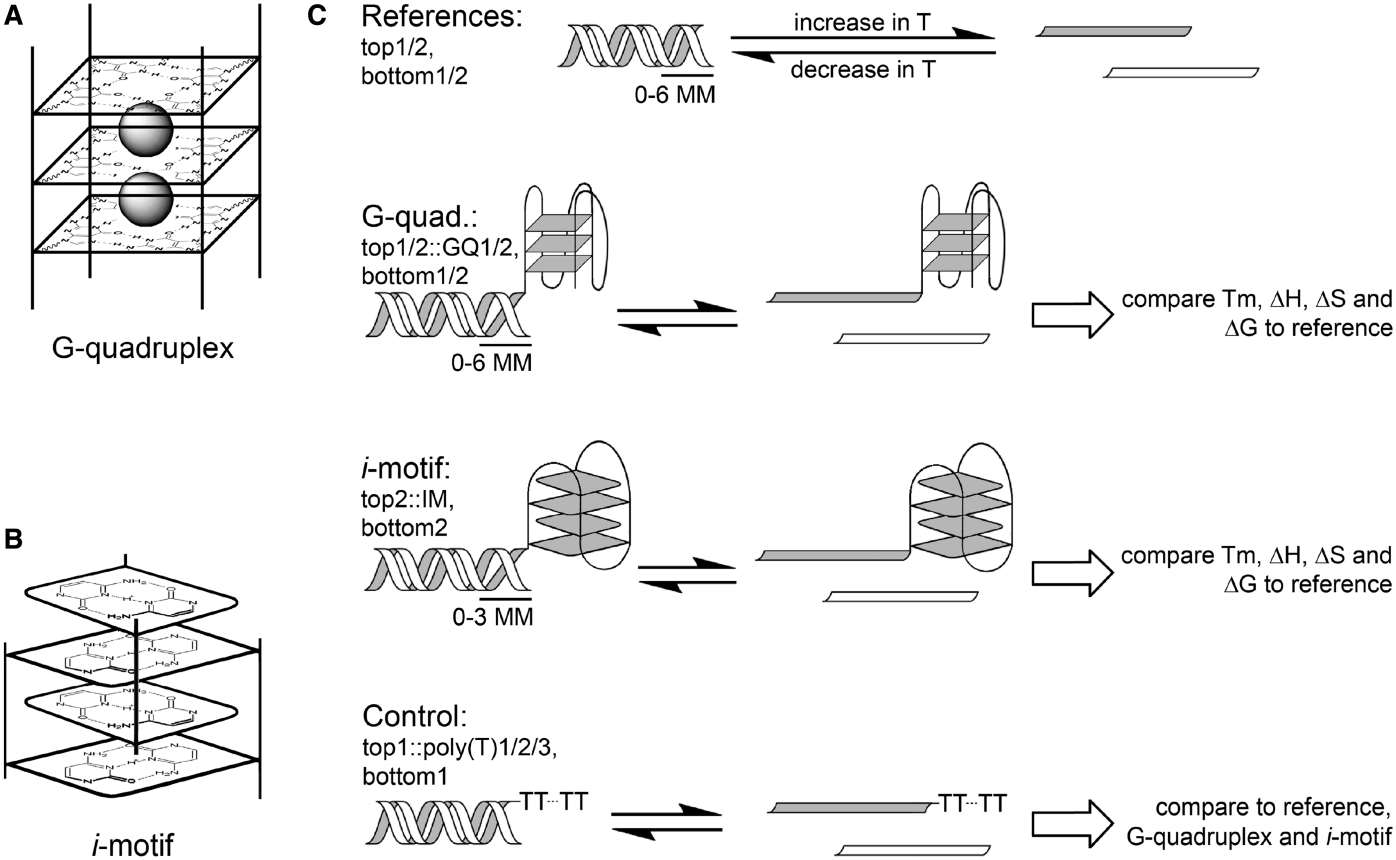
Schemes of the four-stranded structures in the focus of this study and experimental design. (**A**) Schematic of a G-quadruplex. Poly(G) stretches interact via W–C and Hoogsteen hydrogen bonds to form G-tetrads (squares) capable of stacking together. Quadruplex thermodynamic stability is enhanced by mono- and divalent cations (grey spheres). (**B**) Schematic of an *i-*motif: Poly(C) pairs interact in protic environments (squares) and stack together in an intercalated manner. (**C**) Characterization of duplex stability in various molecular environments. References: Two different double-strands (top1, bottom1 and top2, bottom2) are denatured when they are subjected to high temperature and re-associate at decreasing temperature. Mismatches (MM) decrease thermal stability and are introduced to induce structural flexibility and one end of the duplex. G-quadruplex, *i*-motif: Thermal melting with two different G-quadruplex-forming sequences (GQ1 and GQ2) and an IM sequence directly joined to one of the duplex strands. The thermodynamic parameters associated with duplex dissociation were subsequently compared with the reference. Control: Poly(T) single-stranded overhangs of varying length (poly(T)1, poly(T)2, poly(T)3) are appended to the duplex to characterize the effect of an unstructured overhang on duplex thermodynamics.

The canonical Watson–Crick (W–C) double-helix conformation of DNA has been extensively studied and is well understood. It is less well known that nucleic acids can also form alternative conformational structures, such as G-quadruplexes and *i*-motifs. A G-quadruplex conformer consists of stacks of G-quartets, which are composed of four guanine bases arranged in a plane and stabilised by Hoogsteen (H) and W–C hydrogen-bonding interactions (1). Enhanced electrostatic and π–π-bonding interactions cause these G-quartets to stack together into G-quadruplex conformers: this stacking has been found to be strongly favoured by the presence of mono- and divalent cations (Figure 1A) (2,3). A wide variety of G-quadruplex topologies have been demonstrated to exist, each varying with regard to the number of guanines involved, relative strand orientation and loop topology (4,5). G-quadruplex stability in isolation has been thoroughly studied, and significant influencing factors include the number of stacked G-quartets, G-quadruplex topology and the type of G-quadruplex-binding cation present in solution (6–11). A G-quadruplex is most likely to form in the presence of at least four consecutive G-runs; hence, putative G-quadruplex-forming regions can be predicted by scanning through nucleic acid genetic sequences (12).

Putative G-quadruplex-forming sequences are abundant throughout the genomes of most living organisms and are frequently found in gene promoter regions and in telomeric domains (12). The putative G-quadruplex-forming sequences that lie within regions of potential duplex formation are less likely to form G-quadruplex conformers owing to the slightly enhanced thermodynamic stability of the duplex structure relative to the quadruplex structure under near physiological conditions (13–20).

The complementary structure to a putative G-quadruplex within a duplex region is the *i*-motif, a series of hemi-protonated cytosine pairs that stack together via both π–π and electrostatic interactions in an intercalated manner (Figure 1B) (21,22). The formation of an *i*-motif is strongly pH-dependent and has been demonstrated *in vitr*o, in sequences complementary to known quadruplex-forming domains (22,23). Given that the thermodynamic stability of adjacent duplex DNA is likely to be affected by the formation of directly proximal G-quadruplex and/or *i*-motif substructures, it is of fundamental interest to verify this destabilizing effect and to establish the distance necessary for duplex restabilization under physiological conditions, and hence more stable G-quadruplex/*i-*motif formation.

A series of thermal melting experiments were therefore performed according to previously described methods to determine whether sequences that are predisposed to form G-quadruplexes and/or *i*-motifs destabilize proximal duplex stretches (Figure 1C) (24–27). Van’t Hoff analysis was used to determine Tm, ΔH, ΔS and ΔG° associated with melting of a series of duplex stretches derived from the *c-kit* oncogene promoter sequence (28).

## MATERIALS AND METHODS

### DNA oligonucleotides

The duplex sequences used for these experiments were derived from a *c-kit* promoter fragment situated 110–119 base pairs (bp) upstream from the transcription start site (Table 1). In the human genome, this segment is directly adjacent to the G-quadruplex-forming sequence c-kit87up (28,30). The first G-quadruplex-forming sequence studied (GQ1) is a modified version of the human telomeric repeat d(GGGTTA)_n_, and the second sequence studied (GQ2) is the *Tetrahymena* telomeric repeat d(GGGGTT)_n_ (31). The *i-*motif-forming sequence (IM) is a fragment of the *bcl-2* promoter region (Py39WT) containing 6 C-runs (32). All sequences were selected such that duplex melting occurred at significantly lower temperature than G-quadruplex and/or *i*-motif melting and were ordered from Invitrogen Ltd (Paisley, UK) and Microsynth AG (Balgach, Switzerland). All reagents used were purchased from Sigma-Aldrich (Gillingham, UK; Buchs, Switzerland).

**Table 1.** DNA oligonucleotide sequences used in the melting experiments

Sequence	Explanation	Sequence 5′ → 3′	Extinction coefficient (mM^−1 ^cm^−1^)
top1	*c-kit* promoter fragment	GGC GCG CAG A	108.9
top1::GQ1	G_3_T_2_G_3_ or G_4_T_2_G_4_ appended to the 3′-end of top1	GGC GCG CAG A (G_3_T_2_)_3_G_3_	292.3
top1::GQ2		GGC GCG CAG A (G_4_T_2_)_3_G_4_	335.7
top1::poly(T)1	poly(T) runs of different length appended to the 3′-end of top1	GGC GCG CAG A T_22_	303.5
top1::poly(T)2		GGC GCG CAG A T_10_	197.5
top1::poly(T)3		GGC GCG CAG A T_27_	347.7
bottom1	complement to top1	TCT GCG CGC C	88.8
bottom1-1MM	1–3 mismatches at the 5′-end of the complement to top 1	ACT GCG CGC C	95.7
bottom1-2MM		ATT GCG CGC C	96.9
bottom1-3MM		ATA GCG CGC C	103.8
top2	TA-rich version of top1	GGC GCG CAT AAA A	154.2
top2::GQ2	G_4_T_2_G_4_ appended to the 3′-end of top2	GGC GCG CAT AAA A (G_4_T_2_)_3_G_4_	380.9
top2::IM	*i*-motif appended to the 3′-end of top2	GGC GCG CAT AAA A CAG C_4_GC TC_3_G C_5_T_2_C_2_ TC_3_G CGC_3_ GC_4_T	487.5
bottom2	complement to top2	TTT TAT GCG CGC C	123.4
bottom2-1MM	1–6 mismatches at the 5′-end of the complement to top 2	ATT TAT GCG CGC C	130.3
bottom2-2MM		AAT TAT GCG CGC C	137.2
bottom2-3MM		AAA TAT GCG CGC C	144.1
bottom2-4MM		AAA AAT GCG CGC C	151.0
bottom2-5MM		AAA ATT GCG CGC C	144.1
bottom2-6MM		AAA ATA GCG CGC C	151.0
GQ2::bottom2	G_4_T_2_G_4_ or *i*-motif appended to the 5′-end of bottom2	(G_4_T_2_)_3_G_4_ TTT TAT GCG CGC C	350.2
IM::bottom2		TC_4_G C_3_GC GC_3_T C_2_T_2_C_5_ GC_3_T CGC_4_ GAC TTT TAT GCG CGC C	456.8

Extinction coefficients have been calculated for unpaired oligonucleotides from the corrected values of nucleoside-5′-monophosphates (29).

### UV melting experiments

Temperature-dependent absorption measurements were recorded at 260 and 295 nm on a Cary 300 Bio UV/vis spectrophotometer (Varian Inc., Palo Alto, USA) equipped with a Cary Temperature Controller using Hellma 115B-QS 10 mm precision quartz cuvettes (Hellma GmbH & Co. KG, Müllheim, Germany). DNA samples were diluted with buffer to a consistent concentration of 3µM/strand. Each DNA sample was dissolved in 400 µL of freshly prepared 10 mM Britton-Robinson buffer (3.3 mM H_3_BO_3_, 3.3 mM H_3_PO_4_, 3.3 mM CH_3_COOH, 80 mM KCl, various pH). Each sample was then degassed, overlaid with 150 µL of mineral oil and sealed before measurements to prevent evaporation. Three absorption ramps were recorded in a temperature range of 10–90°C at a temperature changing rate of 0.25°C/min (90°C – 10°C – 90°C – 10°C), followed by van’t Hoff analysis as described previously in the literature (Supplementary Figure S1) (25,33). ΔG values were calculated for standard conditions, *i.e.* T = 298.15 K. By simultaneously monitoring absorption at 295 nm, G-quadruplex/*i*-motif melting was ensured to occur at higher temperatures than duplex melting (26).

Melting experiments were performed with the pairs of oligonucleotides shown in Table 1. To establish duplex stability in the absence of a four-stranded structure, the following sequences were used: top1 paired with bottom1, bottom1-1MM, bottom1-2MM or bottom1-3MM, as well as top2 paired with bottom2, bottom2-1MM, bottom2-2MM, bottom2-3MM, bottom2-4MM, bottom2-5MM or bottom2-6MM (Figure 1C, ‘reference’). Duplex stability in the presence of a G-quadruplex was assessed using top1::GQ1 paired with bottom1, top1::GQ2 paired with bottom1 and top2::GQ2 paired with bottom2, bottom2-1MM, bottom2-2MM, bottom2-3MM, bottom2-4MM, bottom2-5MM or bottom2-6MM (Figure 1C, ‘G-quadruplex’). To characterize duplex stability in the presence of an *i*-motif, the following sequences were used: top2::IM paired with bottom2, bottom2-1MM, bottom2-2MM or bottom2-3MM (Figure 1C, ‘*i*-motif’). Duplex stability in the presence of a G-quadruplex or an *i*-motif at different pH values was established using the following sequences: top2::GQ2 paired with bottom2 and top2::IM paired with bottom2. To investigate duplex stability in the presence of both a G-quadruplex and an *i-*motif, the following sequences were used: top2::IM paired with GQ2::bottom2; top2::GQ2 paired with IM::bottom2. Finally, duplex stability in the presence of an unstructured poly-T overhang was studied using the following sequences: top1::poly(T)1, top1::poly(T)2 or top1::poly(T)3 paired with bottom1 (Figure 1C, ‘Control’).

Measurements were repeated at least three times for each oligonucleotide pair, and the experimental error indicated corresponds to the standard deviation (1σ). Analysis of variance was performed in MATLAB (student version 7.12.0.635) to assess whether a change in duplex thermodynamic stability was significant using the built-in statistics toolbox (34).

## CD melting

Duplex dissociation and annealing was monitored by following ellipticity at 280 nm over a temperature range of 90–15°C using a AP ChiralScan circular dichroism (CD) spectrophotometer (Surrey, UK) and Hellma 100QX cuvettes (Hellma GmbH & Co. KG, Müllheim, Germany) according to a protocol adapted from (24). Oligonucleotides were dissolved in 200 µL 10 mM Tris–HCl, 80 mM KCl (pH 7.40) up to a total concentration of 10 µM per oligonucleotide, pre-heated to 90°C and sealed with a cap before measurements. Two temperature-ellipticity profiles were recorded using a temperature changing rate of 0.30°C/min (90°C −15°C− 90°C), followed by van’t Hoff analysis (25).

Furthermore, CD spectra from 350 to 230 nm were recorded over a temperature range of 90–10°C using a J-810 spectrapolarimeter (Jasco Inc., Japan). Oligonucleotides were dissolved in 10 mM Britton–Robinson buffer at pH 7.4 or 4.0 to reach a total DNA concentration of 3 μM per strand, degassed at 90°C for 10 min, transferred to a 10 mm pathlength cuvette (Hellma GmbH & Co. KG, Müllheim, Germany) and overlaid with mineral oil before the begin of the measurements. Analogous to the UV melting experiments, the temperature changing rate was set to 0.25°C/min.

## RESULTS

### G-quadruplex stability proximal to duplex DNA

**Figure 2.**
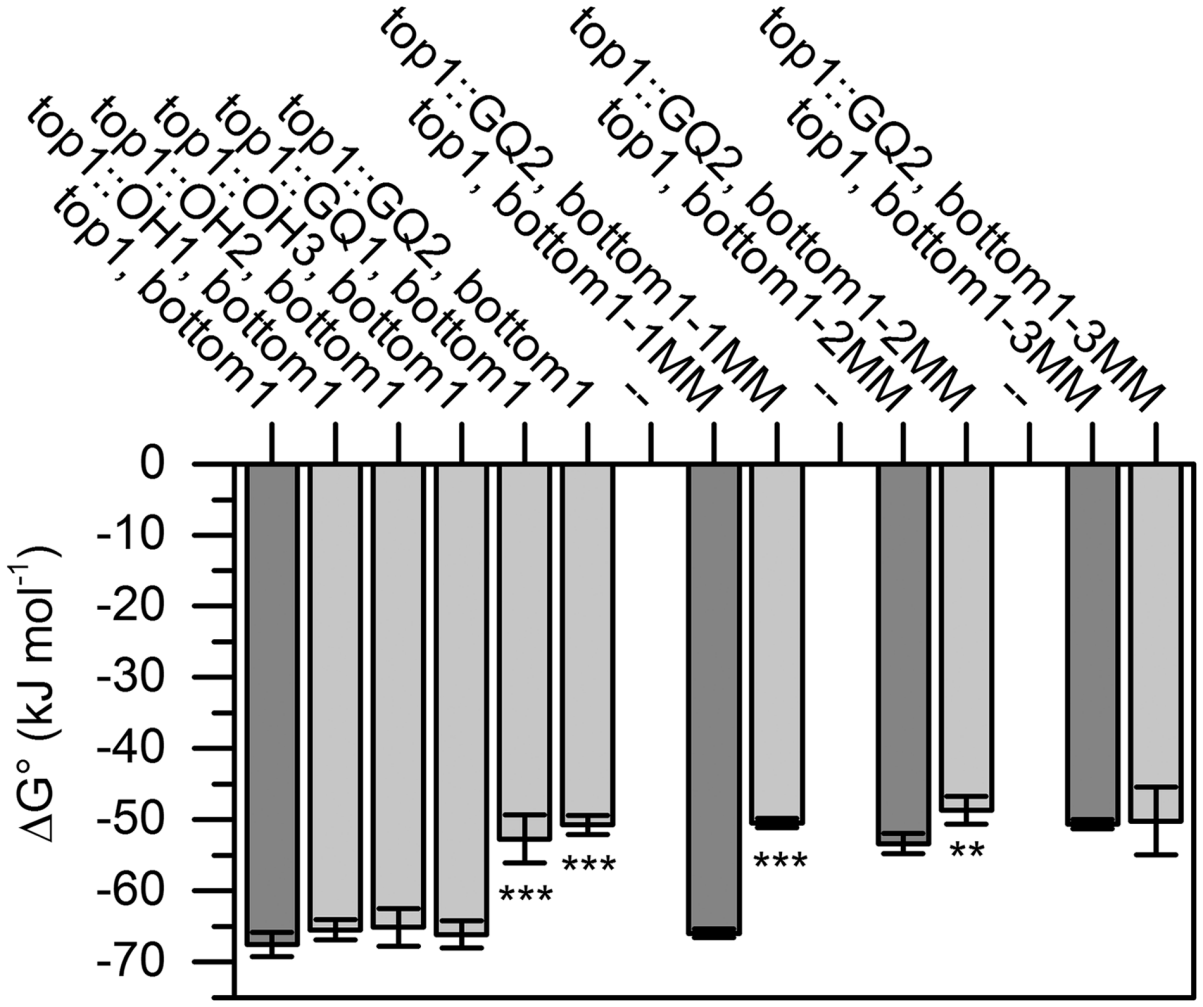
Thermodynamic stability of a duplex derived from the *c-kit* promoter in the presence and in the absence of an overhang. In the absence of a 3′ overhang (top1, bottom1) ΔG° = −67.5 kJ/mol. A 3′-poly(T) overhang decreases thermodynamic stability (ΔG° = −65.0 kJ/mol), but this decrease is not statistically significant (top1::OH1/2/3, bottom1, *P* > 0.05). The presence of a G-quadruplex significantly increases ΔG° (top1::GQ1/2, bottom1). Duplex destabilisation in the presence of a G-quadruplex is significant in the presence of 1 or 2 mismatches immediately proximal to the appended secondary structure motif (top1/top1::GQ2, bottom1-1MM and top1/top1::GQ2, bottom1-2MM). No significant changes are observable in the presence of three mismatches. Statistical significance of changes was estimated by analysis of variance, and Bonferroni correction for multiple testing was applied when necessary. **P < 0.01, ***P < 0.001.

**Figure 3.**
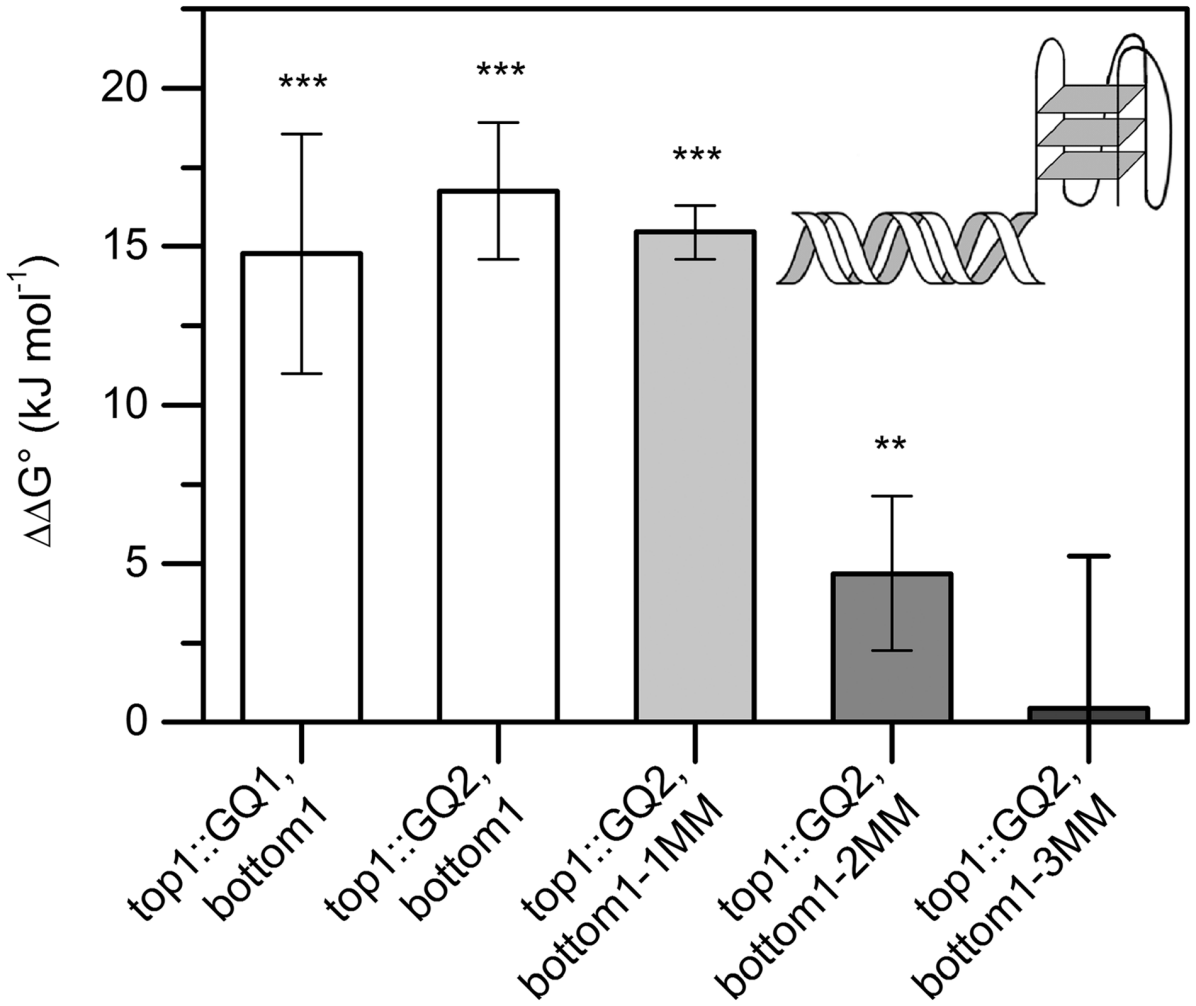
G-quadruplex-induced destabilization of a duplex sequence derived from the *c-kit* promoter. A significant decrease in duplex stability was observed in the presence of a proximal G-quadruplex (top1::GQ1, bottom1 and top1::GQ2, bottom1). Duplex destabilization was observed for up to two mismatches at the vicinity of the G-quadruplex (top1::GQ2, bottom1-1MM and top1::GQ2, bottom1-2MM), but not for three mismatches (top1::GQ2, bottom1-3MM). Data from Figure 2. **P < 0.01, ***P < 0.001.

A number of thermal melting experiments were performed to determine whether and to what extent G-quadruplexes destabilize directly adjacent duplex DNA regions (25,26). Figures 2 and 3 show the results of the first set of sequences that were studied by UV melting experiments. The standard free enthalpy associated with annealing of the control duplex (top1, bottom1) was found to be −67.5 ± 1.7 kJ/mol with a melting temperature of 58.6 ± 0.7°C. The presence of a directly proximal G-quadruplex was observed to decrease the energy required for duplex melting by 14.8 to 16.8 kJ/mol, depending on the nature of the G-quadruplex (Table 2). For both sequence pairs, a smooth transition was observed on duplex melting, which is typical for a two-state transition (Supplementary Figures S1A and S2A). In accord with the experimental design depicted in Figure 1, duplex formation could be confirmed to be a bimolecular process, whereas G-quadruplex formation was unimolecular (Supplementary Figures S1D, S2D and F). CD experiments with top1/bottom1 and top1::GQ2/bottom provided evidence for G-quadruplex formation and minimal overlap between duplex and quadruplex melting (ΔTm >25°C, Supplementary Figure S2A and B). Linear dependence of spectral changes in the relevant temperature range and the results of van’t Hoff plot analysis further indicated that duplex dissociation is a cooperative process under the experimental conditions chosen for these studies (Supplementary Figures S1E, S2C and E).

**Table 2.** Melting temperature of B-DNA in the presence and absence of an adjacent G-quadruplex or *i*-motif as determined by UV thermal melting experiments

Sequence pair	pH	Exp. Tm (°C)	ΔTm (°C)	ΔH (kJ mol^−1^)	ΔS (J K^−1 ^mol^−1^)	ΔG° (kJ mol^−1^)	ΔΔG° (kJ mol^−1^)
top1, bottom1	7.4	58.6 ± 0.7		−336.4 ± 11.5	−902.0 ± 32.9	−67.5 ± 1.7	
top1::GQ1, bottom1	7.4	52.7 ± 2.5	5.9 ± 2.6	−252.2 ± 56.6	−585.0 ± 98.2	−52.7 ± 3.4	14.8 ± 3.8
top1::GQ2, bottom2	7.4	51.4 ± 1.8	7.2 ± 1.9	−213.8 ± 11.0	−547.0 ± 34.8	−50.7 ± 1.3	16.8 ± 2.2
top::poly(T)1, bottom1	7.4	56.9 ± 0.7	1.7 ± 1.0	−332.8 ± 12.1	−896.7 ± 36.4	−65.5 ± 1.4	2.0 ± 2.2
top2, bottom2	7.4	54.3 ± 0.5		−381.2 ± 7.4	−1052.3 ± 23.5	−67.5 ± 0.6	
top2::GQ2, bottom2	7.4	49.9 ± 0.3	4.4 ± 0.6	−245.7 ± 14.6	−648.8 ± 44.8	−52.3 ± 1.2	15.2 ± 1.4
top2, bottom2	4.0	39.4 ± 0.9		−254.9 ± 16.5	−703.9 ± 51.9	−45.1 ± 1.2	
top2::IM, bottom2	4.0	35.2 ± 0.8	4.1 ± 1.2	−254.0 ± 2.1	−720.7 ± 4.3	−39.0 ± 1.0	6.0 ± 1.6

Error indicated as 1σ.

Further thermal melting experiments revealed that the presence of a long poly(T) overhang does not destabilize the adjacent double helix to a significant extent (top1::poly(T)1, top1::poly(T)2, or top1::poly(T)3, paired with bottom1; ΔΔG° = 1.4–2.4 kJ/mol, Figure 2 and Table 2), which demonstrates that the decrease in thermodynamic stability is G-quadruplex specific, and not the result of base overhang destabilization. Mismatches were then introduced at the 5′-end of the complementary strand, and further UV melting experiments were carried out in the presence and absence of a G-quadruplex-forming sequence in an attempt to mitigate the destabilizing effect of the G-quadruplex (Figure 2). A slight decrease in ΔΔG° was observed with introduction of one mismatch (top1::GQ2, bottom1-1MM, ΔΔG° = 15.4 ± 0.9 kJ/mol, Figures 2 and 3), followed by a strong decrease on insertion of two mismatches (top1::GQ2, bottom1-2MM, ΔΔG° = 4.7 ± 2.4, Figures 2 and 3). However, ΔΔG was found to be statistically insignificant in the presence of three mismatches (top1::GQ2, bottom1-3MM, Figures 2 and 3). As the melting temperature of G_3_T_2_G_3_T_2_G_3_T_2_G_3_ was close to the melting temperatures observed for the duplexes assessed herein, only G_4_T_2_G_4_T_2_G_4_T_2_G_4_ was used to determine the range of the destabilization effect to avoid the duplex-quadruplex transition overlap. All results are summarized in the Supplementary Information (Supplementary Table S1) and were confirmed performing CD thermal melting experiments as described (Supplementary Table S2) (24).

**Figure 4.**
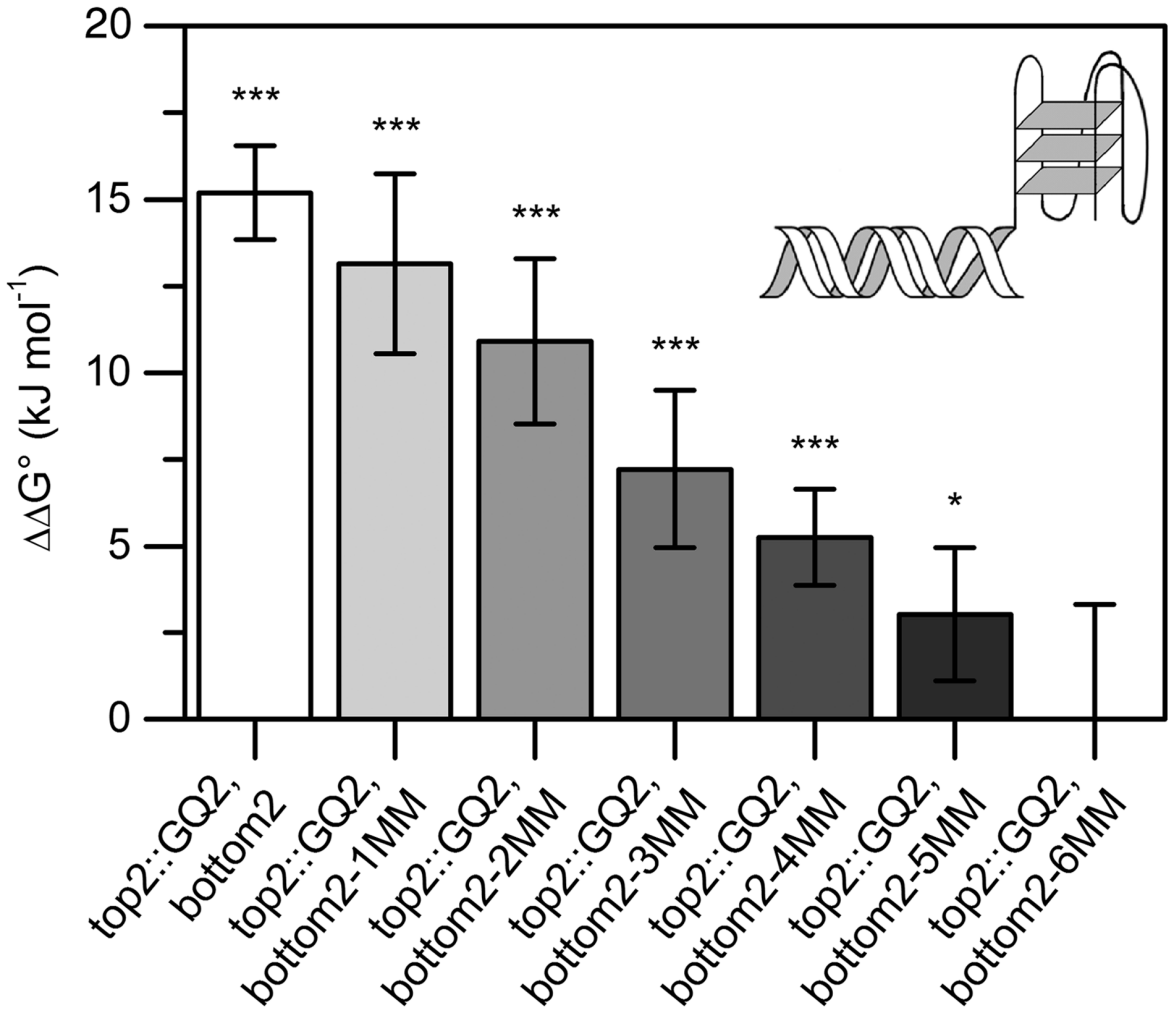
G-quadruplex-induced duplex destabilization of a modified *c-kit* fragment. A G-quadruplex strongly destabilizes the adjacent duplex (top2::GQ2, bottom2). An increasing number of mismatches proximal to the G-quadruplex region results in a significant decrease in ΔΔG° (top2::GQ2, bottom2-1-5MM), approximating zero in the case of six mismatches (top2::GQ2, bottom2-6MM). *P < 0.05, ***P < 0.001.

Similar observations were made for a second set of sequences derived from a modified version of the *c-kit* promoter fragment (Figure 4). The presence of the *Tetrahymena* telomeric G-quadruplex was found to decrease duplex stability by 15.2 kJ/mol (Table 2). As the number of mismatches was increased, duplex ΔΔG° was found to decrease in a regular manner [ΔΔG°(top2::GQ2, bottom2-1MM) = 13.1 ± 2.6 kJ/mol; ΔΔG°(top2::GQ2, bottom2-2MM) = 10.9 ± 2.4 kJ/mol; ΔΔG°(top2::GQ2, bottom2-3MM) = 7.2 ± 2.2 kJ/mol; ΔΔG°(top2::GQ2, bottom2-4MM) = 5.1 ± 1.4 kJ/mol; ΔΔG°(top2::GQ2, bottom2-5MM) = 3.0 ± 1.9 kJ/mol]. On insertion of six mismatches, ΔΔG° was found to approximate zero and become statistically insignificant (top2::GQ2, bottom2-6MM). Statistical analysis was performed in an analogous manner as for the first set of sequences. Again, the *Tetrahymena* G-quadruplex displayed high thermostability (Tm ∼80°C, ΔTm > 25°C) and was shown to be unimolecular, whereas duplex interaction was demonstrated to be an intermolecular process that can be described by a two-state model (Supplementary Figure S3). The results of the second series of melting experiments are summarized in the Supplementary Information (Supplementary Table S3).

### Py39WT *i*-motif stability proximal to duplex DNA

**Figure 5.**
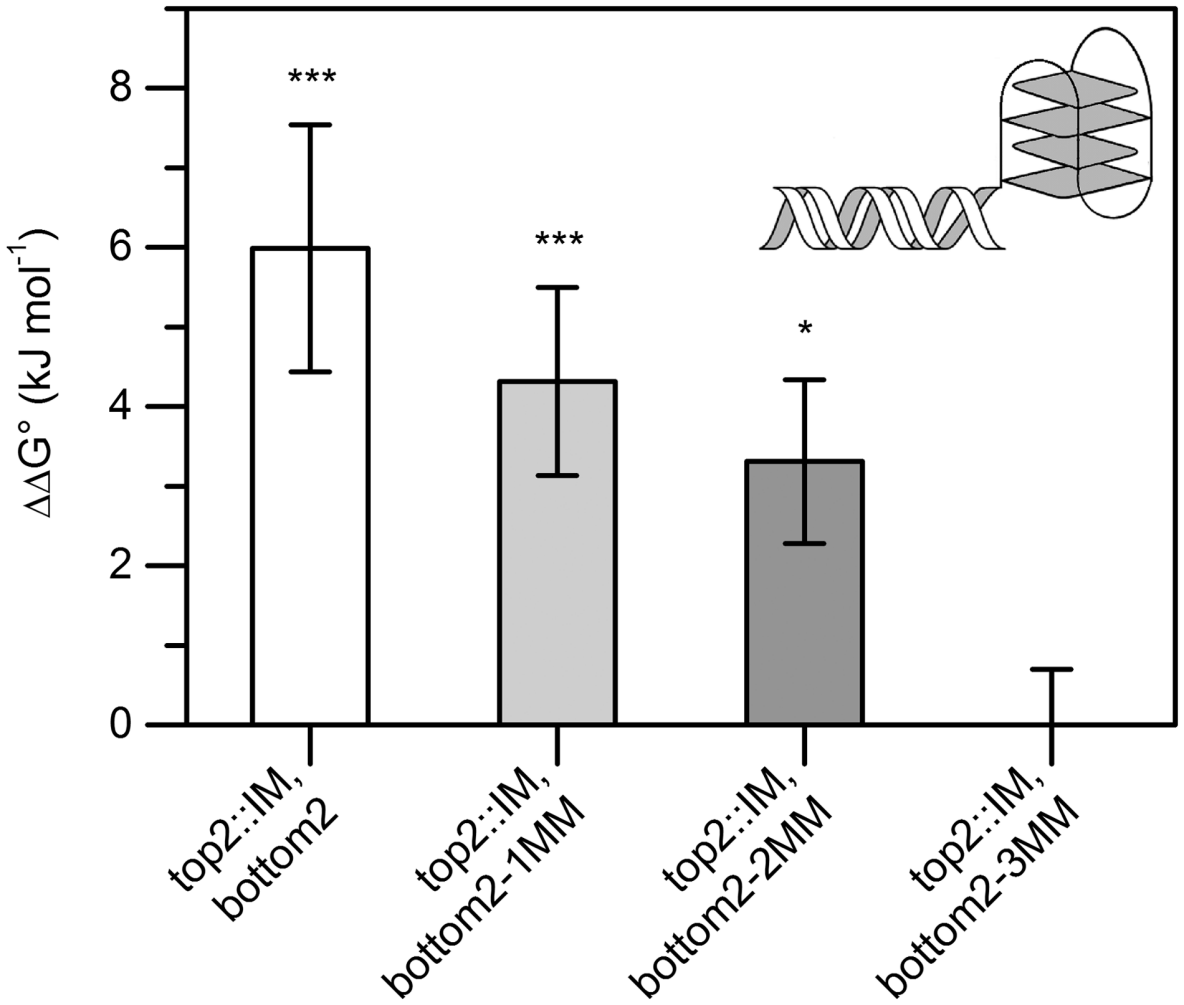
*i*-motif-induced duplex destabilization of a modified *c-kit* fragment. The *i-*motif destabilizes the neighbouring double helix (top2::IM, bottom2). Increasing the number of mismatches proximal to the *i-*motif results in significantly decreasing ΔΔG°, approximating zero on introducing three mismatches at the vicinity to the i-motif (top2::IM, bottom2-3MM). *P < 0.05, ***P < 0.001.

To determine whether formation of a proximal *i*-motif induces destabilization of an adjacent duplex stretch, an additional series of UV melting experiments were performed (Figure 5). Owing to the pH sensitivity and to avoid overlap of duplex and *i*-motif unfolding, these experiments were carried out at pH 4.0 and 4.4, respectively. Attachment of the known IM sequence Py39WT to the 3′-end of C-1B was found to significantly decrease the stability of the adjacent duplex by 6.0 kJ/mol (Table 2). As mismatches were introduced proximal to the *i-*motif-forming region, ΔΔG was found to progressively decrease [ΔΔG° (top2::IM, bottom2-1MM) = 4.3 ± 1.2 kJ/mol; ΔΔG°(top2::IM, bottom2-2MM) = 3.3 ± 1.0 kJ/mol]. After three mismatches had been introduced, ΔΔG° approximated zero (top2::IM, bottom2-3MM). In all cases, melting of the *i*-motif was found to occur at significantly higher temperatures than duplex denaturation (ΔTm > 30°C), and the altered overhang did not seem to affect the unfolding mechanism of the duplex, as it could again be shown to be an intermolecular process that could be approximated by a two-state model (Supplementary Figure S4). All results are summarized in the Supplementary Information (Supplementary Table S4).

### pH dependency and cumulative effects of quadruplex and *i-*motif stability proximal to duplex DNA

**Figure 6.**
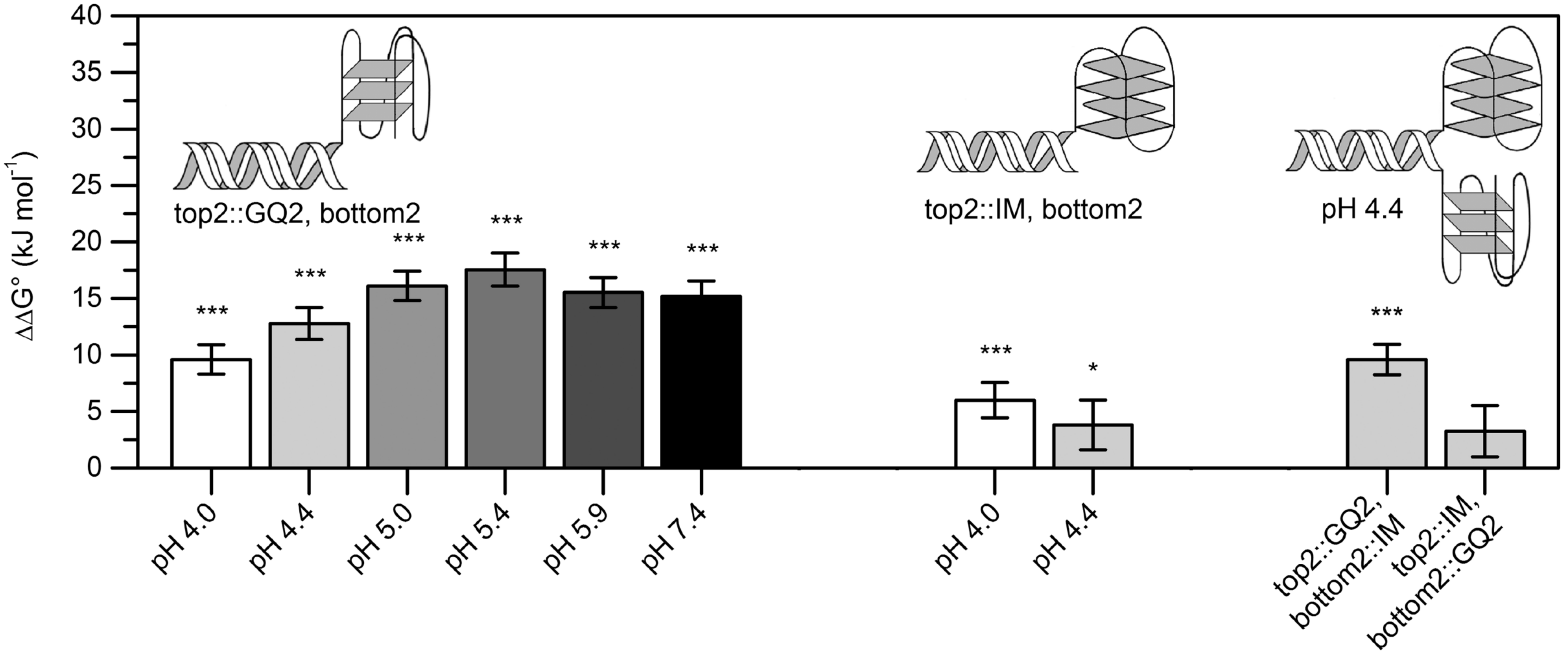
pH dependency of G-quadruplex- and *i-*motif-induced duplex destabilization. Overall destabilization of the duplex DNA adjacent to a G-quadruplex increases with increasing pH (left), whereas duplex destabilization induced by a proximal *i-*motif does not vary significantly with the pH (centre). No cumulative duplex destabilization effects observed when sequences that can form both G-quadruplex and *i-*motif are present (right). *P < 0.05, ***P < 0.001.

A series of UV melting experiments were carried out to determine whether G-quadruplex- and *i*-motif-induced duplex destabilizations are pH dependent (Figure 6). Duplex destabilization induced by the *Tetrahymena* telomeric repeat GQ2 has been found to increase with increasing pH [ΔΔG°(pH 4.0) = 9.6 ± 1.3; ΔΔG°(pH 4.4) = 12.8 ± 1.4; ΔΔG°(pH 5.0) = 16.1 ± 1.3; ΔΔG°(pH 5.4) = 17.5 ± 1.5; ΔΔG°(pH 5.9) = 15.5 ± 1.3; ΔΔG°(pH 7.4) = 15.2 ± 1.4]. Decreases in duplex stability observed in the presence of the Py39WT *i*-motif ranged between 6.0 ± 1.6 kJ/mol and 3.8 ± 2.2 kJ/mol at pH 4.0 and 4.4, respectively. To assess the cumulative effects of quadruplex and *i-*motif conformers on duplex stability, duplex Tm was also measured in the presence of both adjacent G-quadruplex and *i*-motif at pH 4.40 (Figure 5). Any cumulative effects of the combination of both G-quadruplex and *i*-motif-forming regions on duplex destabilization were not observed be any greater than the destabilizing effects of either quadruplex or *i-*motif individually [ΔΔG°(top2::IM, GQ2::bottom2) = 3.3 ± 2.3 kJ/mol; ΔΔG°(top2::GQ2, IM::bottom2) = 9.6 ± 0.7 kJ/mol]. Control experiments support two-state unfolding of the duplex and confirmed the presence of the G-quadruplex, though with decreased thermal stability (Supplementary Figure S5). All results are summed up in the Supplementary Information (Supplementary Table S5). Higher pH values could not be assessed, as duplex melting and *i-*motif dissociation began to overlap in the thermal melting profiles disallowing van’t Hoff analysis (25).

## DISCUSSION

This research demonstrates that both G-quadruplex and *i*-motif substructures cause a distance-dependent destabilization of directly adjacent duplex DNA. At the simplest level, these folded conformations could be regarded as steric blocks capable of pushing proximal duplex strands apart. Indeed, this conceptualization could also offer a rationale for the increased G-quadruplex destabilization of duplex DNA relative to *i*-motif destabilization. The diameters of quadruplex motifs range between 24.3 and 28.7 Å, and the *Tetrahymena* G-quadruplex has been found to possess a diameter of 25.0 Å (35–38). In comparison, *i*-motif diameters range from 14.7 to 19.4 Å (39,40). Given that G-quadruplexes span a diameter ∼25% larger than *i*-motif, it is not surprising that their duplex destabilizing capacity is lower than that observed for G-quadruplex motifs. This difference in stability reflects a stronger contribution from entropy than from enthalpy, although both kinetic and thermodynamic influences are present (Table 2). This means that hydrogen bond formation and base stacking in the duplex are more stable in the absence of a four-stranded structure, and that its entropy is also more favourable. The binding of cations and water molecules to ordered nucleic acids is known to provide a major entropic contribution to their formation (41). These findings are somewhat in contrast to a study conducted by Ren *et al.* (42) who reported on little ‘crosstalk’ between a G-quadruplex and an adjacent poly(A-T) duplex. Even though the G-quadruplex that was used is similar in diameter (∼25.4 Å) (43), the authors designed their construct such that there was an unpaired adenine at the junction between the duplex and the G-quadruplex, which might be sufficient to bridge G-quadruplex-induced destabilization.

Interestingly, the ΔΔG values observed during the melting experiments investigating the cumulative effects of G-quadruplex and *i-*motif formation proximal to a duplex closely approximate the ΔΔG values observed in either the presence of a G-quadruplex or an *i-*motif. These values therefore suggest that G-quadruplex and *i*-motif formation are likely to be mutually exclusive within duplex contexts: either a G-quadruplex or an *i-*motif is able to form, but the formation of both motifs proximal to duplex DNA appears to be energetically unfavourable, at least at lower pH values (44).

Additionally, given that duplex conformation is less stable at lower pH, duplex destabilization would be expected to be of lower absolute value (ΔΔG°). Thus, we also report the calculation of ΔΔG°/ΔG°(duplex alone), taking into account this inherent duplex destabilization at lower pHs to better evaluate any pH influence of quadruplex formation on duplex DNA (Table 3). These values demonstrate that once duplex destabilization at low pHs is accounted for, quadruplex destabilisation of duplex DNA appears to be independent of pH.

**Table 3.** pH dependence of G-quadruplex/*i*-motif-induced destabilization of an adjacent duplex derived from the *c-kit* promoter sequence

Sequence name	pH	Absolute destabilization ΔΔG° (kJ mol^−1^)	Relative destabilization ΔΔG°/ΔG° (%)
top2::GQ2, bottom2	4.0	9.6 ± 1.3	21.28 ± 2.94
	4.4	12.8 ± 1.4	24.06 ± 2.68
	5.0	16.1 ± 1.3	25.39 ± 2.08
	5.4	17.5 ± 1.5	26.52 ± 2.32
	5.9	15.5 ± 1.3	23.31 ± 1.98
	7.4	15.2 ± 1.4	22.52 ± 2.08
top2::IM, bottom2	4.0	6.0 ± 1.6	13.3 ± 3.57
	4.4	3.8 ± 2.2	7.14 ± 4.14

Experimental errors are given in ± 1σ. 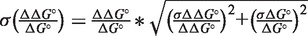.

As many putative G-quadruplex-forming and IM sequences are surrounded by stretches of duplex DNA, these results are of particular interest and could help to address whether these higher-order substructures are likely to form in a physiological context. As the thermodynamic stabilities of duplex and quadruplex substructures are similar, proximity to a quadruplex-forming region and subsequent loss of hydrogen bond stability in the duplex region could result in an equilibrium shift towards duplex conformation to re-establish thermodynamic stability. The concept of G-quadruplexes as regulatory motifs throughout the genome has been proposed to be dependent on neighbouring sequences, although alternative intercellular influences such as chromatin structure, duplex supercoiling and the presence of specific proteins (such as chromatin structure) could also shift the equilibrium to favour quadruplex formation (45–48). The regulatory role of quadruplex structures within the genome remains a challenge to be addressed by the scientific community.

This work represents a step towards that goal and demonstrates a novel phenomenon: both G-quadruplexes and *i-*motifs are capable of destabilizing directly proximal duplex DNA. Further, G-quadruplex-forming and IM sequences should be tested, and a variety of other duplex sequences remain to be assessed. Also, model-free approaches may be used in conjunction with thermal denaturation experiments. Continued investigation would afford quantitative prediction of exactly how proximal to a duplex a quadruplex-forming or IM regions can exist without affecting duplex stability and hence provide a measurable switch for duplex versus quadruplex formation.

## SUPPLEMENTARY DATA

Supplementary Data are available at NAR Online: Supplementary Tables 1–5, Supplementary Figures 1–5, Supplementary Methods and Supplementary References [49–59].

## FUNDING

German Academic Exchange Service [D/09/48185, to S.B.L.K.] and a Forschungskredit of the University of Zurich [57010302, to S.L.B.K.]; a Research Council UK Academic Fellowship (to J.L.H.); State Secretary for Education and Research [COST action CM 1105, to R.K.O.S.]; European Research Council [ERC Starting Grant MIRNA 259092, to R.K.O.S.]; Isaac Newton Trust Research Grant (to A.C.E.). Funding for open access charge: Research Council UK Academic Fellowship, University of Cambridge (to J.L.H.).

*Conflict of interest statement*. None declared.

## Supplementary Material

Supplementary Data

## References

[1] Gellert M, Lipsett MN, Davies DR (1962). Helix formation by guanylic acid. Proc. Natl Acad. Sci. USA.

[2] König SLB, Evans AC, Huppert JL (2010). Seven essential questions on G-quadruplexes. Biomol. Concepts.

[3] Mergny JL, De Cian A, Ghelab A, Saccà B, Lacroix L (2005). Kinetics of tetramolecular quadruplexes. Nucleic Acids Res..

[4] Huppert JL (2008). Four-stranded nucleic acids: structure, function and targeting of G-quadruplexes. Chem. Soc. Rev..

[5] Guédin A, Gros J, Alberti P, Mergny JL (2010). How long is too long? Effects of loop size on G-quadruplex stability. Nucleic Acids Res..

[6] Halder K, Hartig JS, Sigel A, Sigel H, Sigel RKO (2011). Structural and catalytic roles of metal ions in RNA.

[7] Risitano A, Fox KR (2003). The stability of intramolecular DNA quadruplexes with extended loops forming inter- and intra-loop duplexes. Org. Biomol. Chem..

[8] Wang Y, Patel DJ (1993). Solution structure of the human telomeric repeat d[AG3(T2AG3)3] G-tetraplex. Structure.

[9] Kypr J, Kejnovská I, Renčiuk D, Vorlíčková M (2009). Circular dichroism and conformational polymorphism of DNA. Nucleic Acids Res..

[10] Rosu F, Gabelica V, De Pauw E, Antoine R, Broyer M, Dugourd P (2012). UV spectroscopy of DNA duplex and quadruplex structures in the gas phase. J. Phys. Chem. A.

[11] Murat P, Singh Y, Defrancq E (2011). Methods for investigating G-quadruplex DNA/ligand interactions. Chem. Soc. Rev..

[12] Huppert JL, Balasubramanian S (2007). Prevalence of G-quadruplexes in the human genome. Nucleic Acids Res..

[13] Alberti P, Mergny JL (2003). DNA duplex-quadruplex exchange as the basis for a nanomolecular machine. Proc. Natl Acad. Sci. USA.

[14] Deng H, Braunlin WH (1995). Duplex to quadruplex equilibrium of the self-complementary oligonucleotide d(GGGGCCCC). Biopolymers.

[15] Kumar N, Maiti S (2005). The effect of osmolytes and small molecule on quadruplex-WC duplex equilibrium: a fluorescence resonance energy transfer study. Nucleic Acids Res..

[16] Kumar N, Sahoo B, Varun KAS, Maiti S, Maiti S (2008). Effect of loop length variation on quadruplex-Watson Crick duplex competition. Nucleic Acids Res..

[17] Li W, Wu P, Ohmichi T, Sugimoto N (2002). Characterization and thermodynamic properties of quadruplex/duplex competition. FEBS Lett..

[18] Miura T, Thomas GJ (1995). Structure and dynamics of interstrand guanine association in quadruplex telomeric DNA. Biochemistry.

[19] Ying L, Green JJ, Li H, Klenerman D, Balasubramanian S (2003). Studies on the structure and dynamics of the human G quadruplex by single-molecule fluorescence energy transfer. Proc. Natl Acad. Sci. USA.

[20] Li W, Miyoshi D, Nakano SI, Sugimoto N (2003). Structural competition involving G-quadruplex DNA and its complement. Biochemistry.

[21] Brazier J, Brown GD (2012). I-motif formation in gene promoters: Unusually stable formation in sequences complementary to known G-quadruplexes. Chem. Comm..

[22] Gehring K, Leroy JL, Guéron M (1993). A tetrameric DNA structure with protonated cytosine-cytosine base pairs. Nature.

[23] Lieblein AL, Buck J, Schlepckow K, Fürtig B, Schwalbe H (2012). Time-resolved NMR spectroscopic studies of DNA i-motif folding reveal kinetic partitioning. Angew. Chem. Int. Ed..

[24] Mandel R, Fasman GD (1974). Thermal denaturation of DNA and DNA:polypeptide complexes. Simultaneous absorption and circular dichroism measurements. Biochem. Bioph. Res. Commun..

[25] Mergny JL, Lacroix L (2003). Analysis of thermal melting curves. Oligonucleotides.

[26] Mergny JL, Lacroix L (2009). UV Melting of G-quadruplexes. Curr. Protoc. Nucleic Acid Chem..

[27] Karsisiotis AI, Hessari NM, Novellino E, Spada GP, Randazzo A, Webba da Silva M (2011). Topological characterization of nucleic acid G-quadruplexes by UV absorption and circular dichroism. Angew. Chem. Int. Edit..

[28] Raiber EA, Kranaster R, Lam E, Nikan M, Balasubramanian S (2012). A non-canonical DNA structure is a binding motif for the transcription factor SP1 *in vitro*. Nucleic Acids Res..

[29] Cavaluzzi MJ, Borer PN (2004). Revised UV extinction coefficients for nucleoside-5′-monophosphates and unpaired DNA and RNA. Nucleic Acids Res..

[30] Todd AK, Haider SM, Parkinson GN, Neidle S (2007). Sequence occurrence and structural uniqueness of a G-quadruplex in the human c-kit promoter. Nucleic Acids Res..

[31] Tran PLT, Mergny JL, Alberti P (2011). Stability of telomeric G-quadruplexes. Nucleic Acids Res..

[32] Kendrick S, Akiyama Y, Hecht SM, Hurley LH (2009). The i-motif in the bcl-2 P1 promoter forms an unexpectedly stable structure with a unique 8:5:7 loop folding pattern. J. Am. Chem. Soc..

[33] Kruschel D, Sigel RKO (2008). Divalent metal ions promote the formation of the 5′-splice site recognition complex in a self-splicing group II intron. J. Inorg. Biochem..

[34] Quinn GP, Keough MJ (2011). Experimental Design and Data Analysis for Biologists.

[35] Amrane S, Adrian M, Heddi B, Serero A, Nicolas A, Mergny JL, Phan AT (2012). Formation of pearl-necklace monomorphic G-quadruplexes in the human CEB25 minisatellite. J. Am. Chem. Soc..

[36] Do NQ, Lim KW, Teo MH, Heddi B, Phan AT (2011). Stacking of G-quadruplexes: NMR structure of a G-rich oligonucleotide with potential anti-HIV and anticancer activity. Nucleic Acids Res..

[37] Heddi B, Phan AT (2011). Structure of human telomeric DNA in crowded solution. J. Am. Chem. Soc..

[38] Wang Y, Patel DJ (1993). Solution structure of a parallel-stranded G-quadruplex DNA. J. Mol. Biol..

[39] Esmaili N, Leroy JL (2005). i-motif solution structure and dynamics of the d(AACCCC) and d(CCCCAA) tetrahymena telomeric repeats. Nucleic Acids Res..

[40] Canalia M, Leroy JL (2009). [5mCCTCTCTCC]4: an i-motif tetramer with intercalated T-T pairs. J. Am. Chem. Soc..

[41] Lu M, Guo Q, Kallenbach NR (1993). Thermodynamics of G-tetraplex formation by telomeric DNAs. Biochemistry.

[42] Ren J, Qu X, Trent JO, Chaires JB (2002). Tiny telomere DNA. Nucleic Acids Res..

[43] Parkinson GN, Lee MPH, Neidle S (2002). Crystal structure of parallel quadruplexes from human telomeric DNA. Nature.

[44] Dhakal S, Yu Z, Konik R, Cui Y, Koirala D, Mao H (2012). G-quadruplex and i-motif are mutually exclusive in ILPR double-stranded DNA. Biophys. J..

[45] Arora A, Nair DR, Maiti S (2009). Effect of flanking bases on quadruplex stability and Watson-Crick duplex competition. FEBS J..

[46] Fang G, Cech TR (1993). The beta subunit of Oxytricha telomere-binding protein promotes G-quartet formation by telomeric DNA. Cell.

[47] Gray JT, Celander DW, Price CM, Cech TR (1991). Cloning and expression of genes for the Oxytricha telomere-binding protein: Specific subunit interactions in the telomeric complex. Cell.

[48] Ou TM, Lu YJ, Tan JH, Huang ZS, Wong KY, Gu LQ (2008). G-quadruplexes: targets in anticancer drug design. ChemMedChem.

[49] Leroy JL, Gueron M, Mergny JL, Helene C (1994). Intramolecular folding of a fragment of the cytosine—rich strand of telomeric DNA into an i-motif. Nucleic Acids Res..

[50] Mergny JL, Phan AT, Lacroix L (1998). Following G-quartet formation by UV spectroscopy. FEBS Lett..

[51] Li X, Peng Y, Ren J, Qu X (2006). Carboxyl modified single-walled carbon nanotubes selectively induce human telomeric i-motif formation. Proc. Natl Acad. Sci. USA.

[52] Turner DH, Bloomfield VA, Crothers DM, Tinoco IJ (2000). Nucleic acids: structures, properties, and functions.

[53] Smith JD, Cappa CD, Wilson KR, Cohen RC, Geissler PL, Saykally RJ (2005). Unified description of temperature-dependent hydrogen bond rearrangements in liquid water. Proc. Natl Acad. Sci. USA.

[54] Damerla RR, Knickelbein KE, Kepchia D, Jackson A, Armitage BA, Eckert KA, Opresko PL (2010). Telomeric repeat mutagenicity in human somatic cells is modulated by repeat orientation and G-quadruplex stability. DNA Repair.

[55] Vorličkova M, Kejnovska I, Sagi J, Renčiuk D, Bednařova K, Motlova J, Kypr J (2012). Circular dichroism and guanine quadruplexes. Methods.

[56] Chen YH, Yang JT, Martinez HM (1972). Determination of the secondary structures of proteins by circular dichroism and optical rotatory dispersion. Biochemistry.

[57] Bucek P, Jaumot J, Avino A, Eritja R, Gargallo R (2009). pH-modulated Watson—Crick duplex-quadruplex equilibria of guanine rich and cytosine-rich DNA sequences 140 base pairs upstream of the c-kit transcription initiation site. Chem. Eur. J..

[58] Dominguez-Martin A, Johannsen S, Sigel A, Operschall BP, Song B, Sigel H, Okruszek A, Gonzalez-Perez JM, Gutierrez-Niclos J, Sigel RKO (2013). Intrinsic acid-base properties of a hexa-2′-deoxynulceoside pentaphosphate, d(ApGpGpCpCpT). Neighboring effects and isomeric equilibria. Chem. Eur. J..

[59] Datta K, Johnson NP, von Hippel PH (2010). DNA conformational changes at the primer-template junction regulate the fidelity of replication by DNA polymerase. Proc. Natl Acad. Sci. USA.

