# Supplementary material for: Distance-dependent duplex DNA destabilization proximal to G-quadruplex/i-motif sequences
Source: Nucleic Acids Res. 2013 Jun 14;41(15):7453–61. doi: 10.1093/nar/gkt476 (PMC3753619; doi:10.1093/nar/gkt476)
Supplement: Supplementary Data [file supp_41_15_7453__index.html]

Distance-dependent duplex DNA destabilization proximal to G-quadruplex/i-motif sequences — Distance-dependent duplex DNA destabilization proximal to G-quadruplex/i-motif sequences — Supplementary Data 

# Distance-dependent duplex DNA destabilization proximal to G-quadruplex/*i*-motif sequences

## Supplementary Data

files

**Files in this Data Supplement:**

- Supplementary Data - pdf file
