# Supplementary material for: Distance-dependent duplex DNA destabilization proximal to G-quadruplex/i-motif sequences
Source: Nucleic Acids Res. 2013 Jun 14;41(15):7453–61. doi: 10.1093/nar/gkt476 (PMC3753619; doi:10.1093/nar/gkt476)
Supplement: Supplementary Data [file supp_gkt476_nar-00608-f-2013-File002.pdf]

SUPPLEMENTARY INFORMATION

Distance-Dependent Duplex DNA Destabilisation Proximal to G-Quadruplex/*i*-Motif Sequences

Sebastian L.B. König [1, 2], Julian L. Huppert[1], Roland K.O. Sigel[2], Amanda C. Evans [1, 3*]

1) Cavendish Laboratory, University of Cambridge, JJ Thomson Avenue, Cambridge CB3 0HE, United Kingdom

2) Institute of Inorganic Chemistry, University of Zurich, Winterthurerstrasse 190, 8057 Zurich, Switzerland

3) University of Nice-Sophia Antipolis, UMR 7272 CNRS, Institut de 40 Chimie de Nice, 28 Avenue Valrose, 06108 Nice, France.

* To whom correspondence should be addressed.

Tel: +33(0)626630739; Fax: +33(0)492076151; Email: amanda.evans@unice.fr

Temperature-dependent absorption profiles. Absorption was monitored at 260 nm and 295 nm over a temperature gradient to assess duplex, G-quadruplex, and *i*-motif thermal stabilities (Figures S1A, S2A, S3A, S4A) (1,2). In all cases, duplex melting could be identified by an increase in absorbance at 260 nm (and slightly at 295 nm) upon thermal denaturation of the duplex. Complete duplex melting was sometime observed to induce a change in baseline, because the molecular environment adjacent to the G-quadruplex is altered. The G-quadruplex was found to be highly stable, and the onset of its dissociation was accompanied by a strong hypochromic shift at 295 nm, while absorption at 260 nm increased slightly as precedented (2). In turn, *i*-motif melting was characterised by a hyperchromic shift and occurred at high temperature depending on the acidity of the buffer (3). At pH 4.0, its melting temperature was found to be 73.5 °C (σ = 1.0, n = 9), which is in excellent agreement with previous reports (Tm = 69.4 °C at pH 4.4) (4). In all cases, the resulting melting curves were fully reversible, *i.e.* heating and cooling curves superimposed well and hysteresis was negligible if present at all. This indicates that at the rate of temperature change used (0.25 °C/min), folding and unfolding reaches equilibrium.

Concentration-dependent UV melting experiments. Total strand concentration in UV melting profiles was varied between 1 μM and 10 μM. Increasing DNA concentration led to an increase in Tm of the duplex, while the thermal stability of the G-quadruplex and the *i*-motif remained unaltered (Figures S1D; S2D,F; S3D,F; S4D,F). This concentration dependence of duplex stability proves that its formation is a bimolecular process. In turn, both the G-quadruplex and the *i*-motif only form from one strand, which is consistent with the experimental design depicted in Figure 1 in the main text. It is important to note that experiments at a total strand concentration of 6 μM were repeated in order to be consistent. Minor deviations within the accuracy of the method were observed in some cases, but the replicates originating from different stock solutions measured on different instruments were generally found to be in excellent agreement (5).

Van't Hoff plots. Van't Hoff analysis requires duplex formation and dissociation to be theoretically described by a two-state model, which assumes that that the two strand are either maximally paired or entirely dissociated (6). Other than by performing van't Hoff analysis, thermodynamic parameters can as well be determined from a plot of 1/Tm versus ln(total strand concentration/4) (Equation 1) (6):

$$\frac{1}{T_m} = \frac{R ln(\frac{c_{tot}^{DNA}}{4})}{\Delta H°} + \frac{\Delta S°}{\Delta H°} \qquad \textbf{(1)}$$

As expected, these plots were linear in all cases, allowing for determination of ΔH° and ΔS° from the slope and the intercept (Figures S1E, S2E, S3E, S4E). The resulting thermodynamic parameters were always within 10 % difference, suggesting that the two-state model is valid.

Temperature-dependent CD spectra. CD spectra were recorded at different temperatures in 80 mM KCl, 10 mM Britton-Robinson buffer and in the presence of a consistent total strand concentration of 6 μM. In order to properly characterise both the duplex system and the quadruplex system (where present, N = 2 - see Equation 2), spectral measurements obtained for a blank sample containing only dissolved salt and buffer were subtracted from the raw ellipticity data. The resulting spectra revealed the existence of an isoelliptic point in the relevant temperature range (Figures S1E, S2C, S3C, S4C). Analyses of the CD spectra suggest that the contributions of the single-strands and the double-stranded DNA to the overall spectrum are linearly dependent over the entire region of wavelengths investigated, further supporting the two-state hypothesis (7).

Subtraction of blank measurements containing both buffer and the duplex-forming strand from raw ellipticity spectra were used to elucidate the contribution of the four-stranded structure to the overall spectrum at different temperatures (Figures S2B, S3B, S4B, N = 1, see below). In the case of the G-quadruplex, a strong positive band around 295 nm and at 260 nm appeared at 70 °C and lower was observed that is consistent with the melting profiles and with the previously reported high thermodynamic stability of the *Tetrahymena* G-quadruplex (8),

which is slightly decreased through the duplex overhang (9). The signature observed in the CD spectra was found to be in good agreement with the spectrum reported for $(T_2G_4)_4$ alone (*Tetrahymena* G-quadruplex with two additional T bases 5' of the G-quadruplex) and may be attributed to a 3+1 antiparallel topology (10).In the case of the *i*-motif, a strong positive peak was observed around 280 nm and a negative peak around 260 nm was observed at 70 °C and lower, in excellent agreement with the literature precedent (4). CD in all figures are expressed as the mean nucleoside residue ellipticity [θ] and have been calculated using:

$$[\theta] = \frac{\theta}{10 * (\sum_{i=1}^{N} n_i * c_i^{DNA}) * l} \; [deg\ cm^2\ dmol^{-1}] \qquad \textbf{(2)}$$

where θ denotes the ellipticity in mdeg (the instrument output), N is the total number of strands contributing to the observed signal, n_i is the number of nucleoside residues, c_i is the strand concentration, and l is the pathlength of the cuvette (11).

pH-dependent UV melting profiles and CD spectra. At pH 4.0, the thermal stability of the G-quadruplex was found to be significantly decreased (Figure S5). Interestingly, this phenomenon was not observed at pH 4.4. G-quadruplex topology and stability have previously been reported to be insensitive to pH (12). As the sequences used involved G-quadruplexes in isolation, the observed quadruplex destabilisation is most likely due to protonation of adenine N1 ($pKa(N1H^+) = 3.71$) and/or cytosine N3 ($pKa(N3H^+) = 4.31 \pm 0.07$) within the sequences adjacent to the G-quadruplex (13).

Supplementary Figures

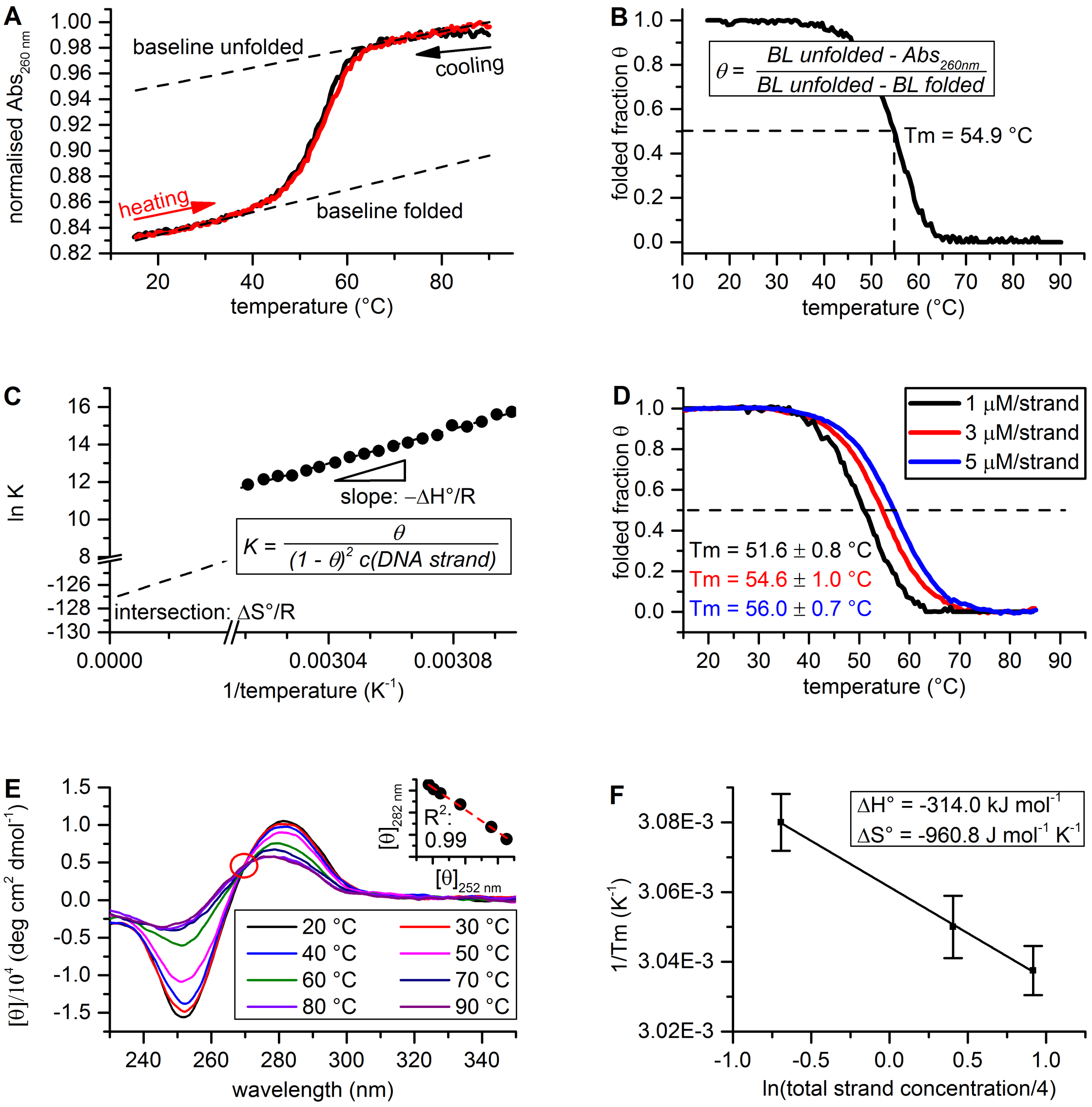

Supplementary Figure S1. *Duplex thermal melting curves and van't Hoff analysis of the sequence pair top1 and bottom1.* (A) Absorption at 260 nm monitored while cooling from 90 °C to 15 °C (black) and re-heating to 90 °C (red). Lower baseline corresponds to the folded duplex, higher baseline corresponds to dissociated single strands. Transition towards the unfolded form is characterised by a pronounced hyperchromic shift. (B) Prevalence of duplex at different temperatures as determined from the heating curve shown in S1A. Melting temperature Tm corresponds to temperature at which 2*c(dsDNA) = c(ssDNA). (C) Extraction of thermodynamic parameters: ln$K$ was plotted against 1/T and -ΔH/R and ΔS/R were extracted from the resulting graph. Equilibrium constant K was calculated as depicted. (D) Concentration-dependence of duplex stability suggests that duplex formation is a bimolecular process. (E) Temperature-dependent CD spectra show the existence of an isoelliptic point (red circle). Both the shape and the position of the isodichroic point is in excellent agreement with CD data describing duplex dissociation (14). Inset: Molar ellipticity at 282 nm vs. molar ellipticity at 252 nm. (F) Thermodynamic analysis of experimental data shown in (D), with thermodynamic parameters in good agreement with the results of the van't Hoff analysis (Supplementary Table S1).

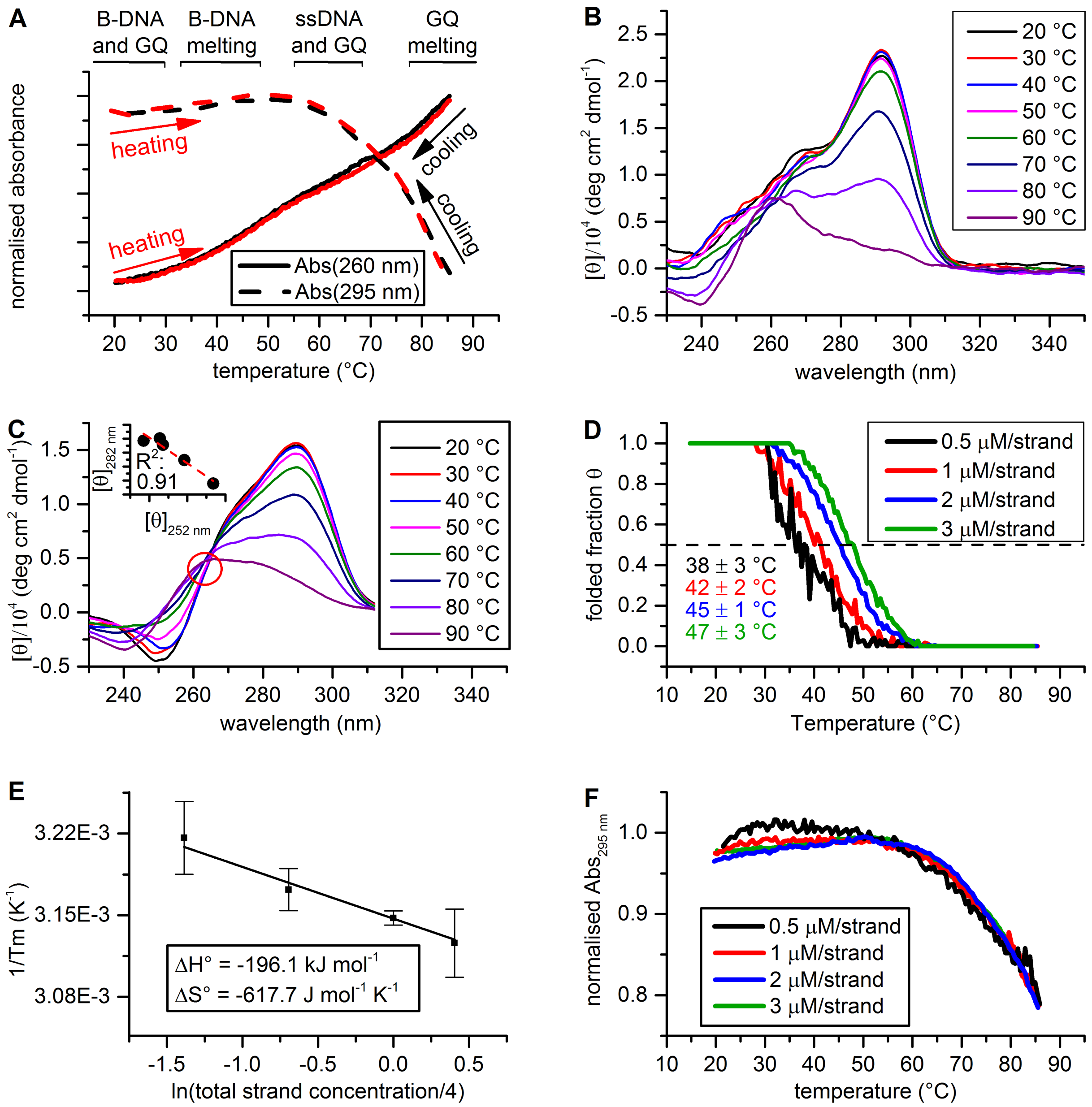

Supplementary Figure S2. *Thermal melting curves and temperature-dependent CD spectra of the sequence pair top1::GQ2 and bottom1.* (A) Absorption at 260 nm (top) and 295 nm (bottom) throughout one entire cooling (black) and reheating cycle (red) following formation and dissociation of both duplex and G-quadruplex DNA. (B) Temperature-dependent CD spectra after subtraction of duplex and buffer contribution. Positive bands appearing around 260 and 295 nm suggest the onset of G-quadruplex formation at approximately 80 °C. (C) Temperature-dependent CD spectra after subtraction of the buffer contribution. The isoelliptic point in the relevant temperature range (60 °C - 20 °C, red circle and inset) suggests that only the population of duplex and single strand change in response to alterations in temperature. Inset: Molar ellipticity at 282 nm vs. molar ellipticity at 252 nm (20 °C ≤ T ≤ 50 °C). (D) Concentration-dependence of duplex stability suggests that its formation is a bimolecular process. (E) Thermodynamic analysis of the experimental data shown in (D). The resulting thermodynamic parameters are in good agreement with the results of the van't Hoff analysis (Supplementary Table S1). (F) Normalised absorption profiles recorded at different strand concentrations superimpose well, suggesting that G-quadruplex formation is a unimolecular process.

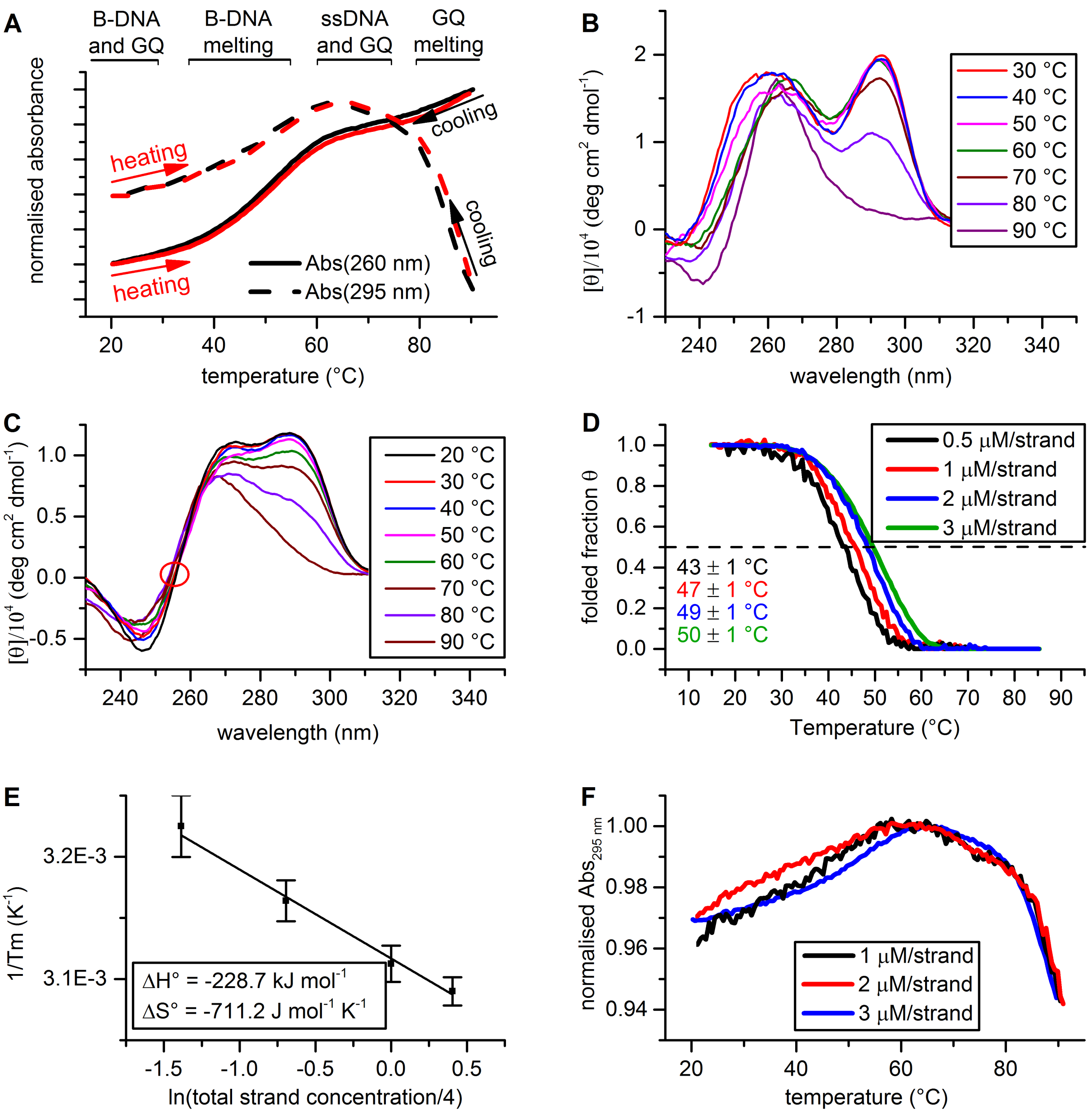

Supplementary Figure S3. *Thermal melting curves and temperature-dependent CD spectra of the sequence pair top2::GQ2 and bottom2.* (A) Absorption at 260 nm (top) and 295 nm (bottom) throughout one entire cooling (black) and reheating cycle (red) following formation and dissociation of both duplex and G-quadruplex DNA. (B) Temperature-dependent CD spectra after subtraction of both duplex and buffer contribution. Positive bands appear around 260 and 295 nm, suggesting the onset of G-quadruplex formation at approximately 80 °C. (C) Temperature-dependent CD spectra after subtraction of the buffer contribution. The isoelliptic point in the relevant temperature range (60 °C - 20 °C, red circle and inset) suggests that only the population of duplex and single strand change in response to alterations in temperature. (D) Concentration-dependence of duplex stability suggests that its formation is a bimolecular process. (E) Thermodynamic analysis of the experimental data shown in (D). The resulting thermodynamic parameters are in good agreement with the results of the van't Hoff analysis (Supplementary Table S3). (F) Normalised absorption profiles recorded at different strand concentrations superimpose well, suggesting that G-quadruplex formation is a unimolecular process.

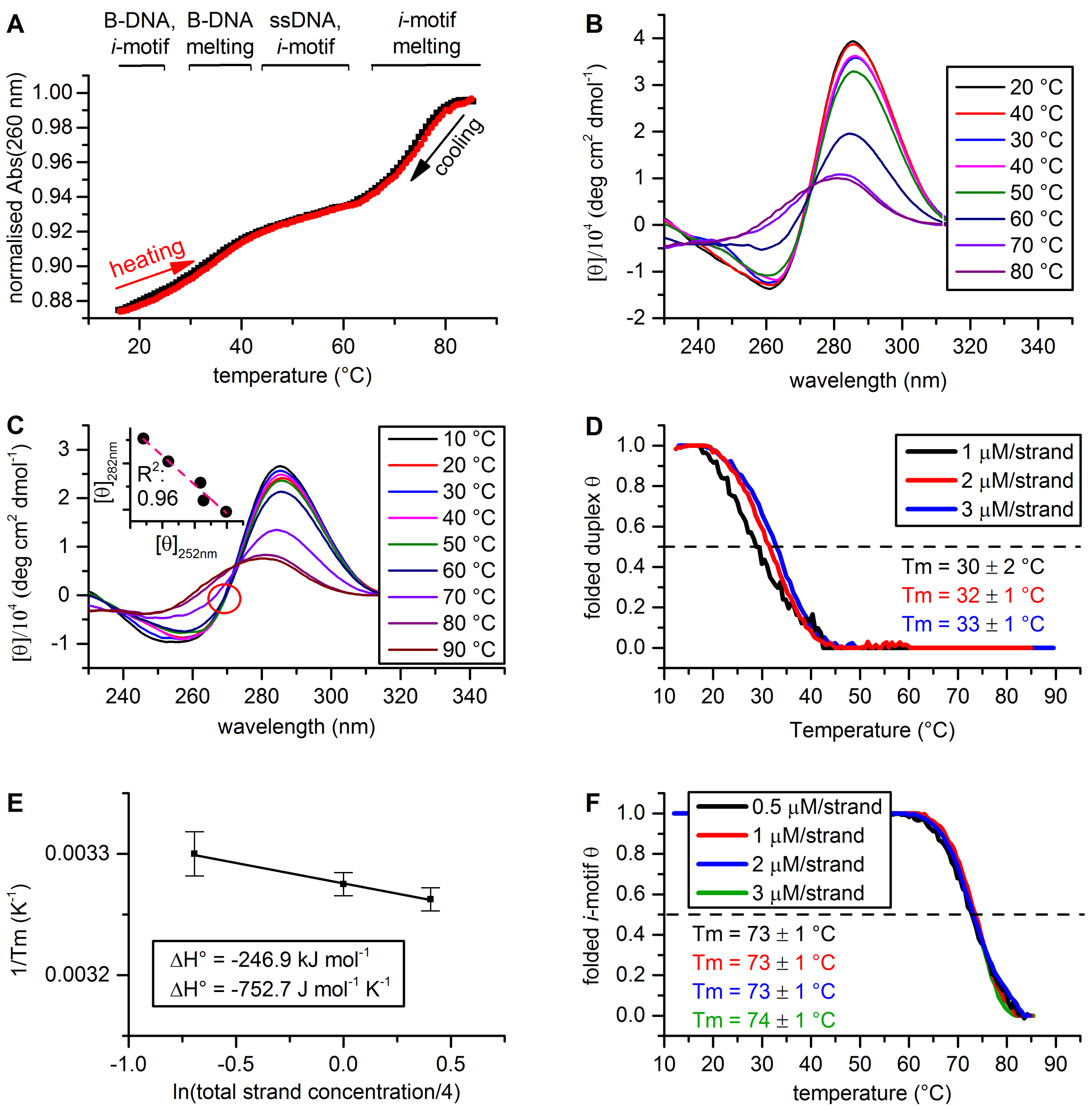

Supplementary Figure S4. *Thermal melting curves and temperature-dependent UV and CD spectra of the sequence pair top2::IM and bottom2.* (A) Absorption at 260 nm followed over a full cooling (black) and reheating cycle (red) following formation and dissociation of both duplex and *i*-motif DNA. (B) Temperature-dependent CD spectra after subtraction of both duplex and buffer contribution. A positive bands around 280 nm and a negative band around 260 nm appear while the temperature is decreased, suggesting the formation of the *i*-motif at approximately 70 °C. (C) Temperature-dependent CD spectra after subtraction of the buffer contribution. The isoelliptic point in the relevant temperature range (60 °C - 20 °C, red circle) suggests that only the population of duplex and single strand change in response to alterations in temperature. Inset: Molar ellipticity at 282 nm vs. molar ellipticity at 252 nm (10 °C ≤ T ≤ 50 °C). (D) Concentration-dependence of duplex stability suggests that its formation is a bimolecular process. (E) Thermodynamic analysis of the experimental data shown in (D). The resulting thermodynamic parameters are in excellent agreement with the results of the van't Hoff analysis (Supplementary Table S4). (F) i-motif stability is concentration independent, suggesting that its formation occurs intramolecularly.

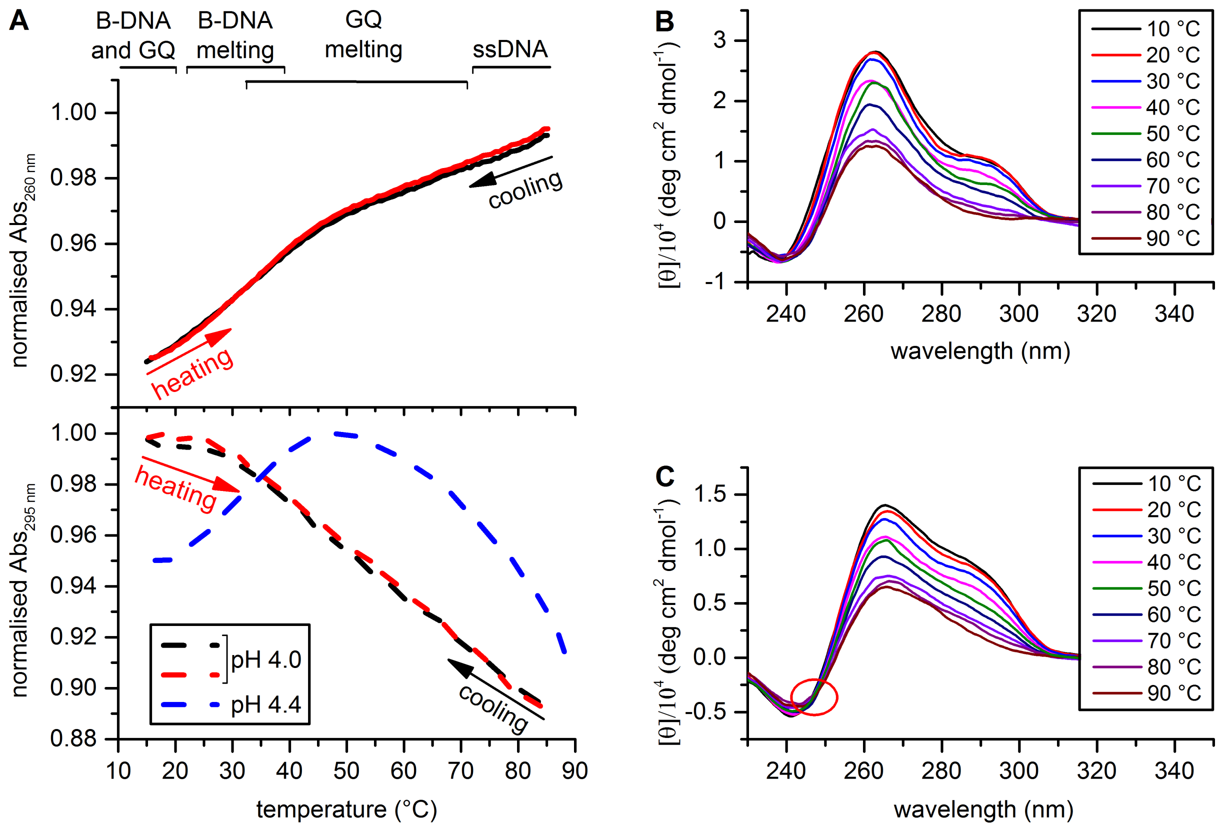

Supplementary Figure S5. *Thermal melting curves and temperature-dependent CD spectra of the sequence pair top2::GQ2 and bottom2 at pH 4.0.* (A) Absorption at 260 nm (top) and 295 nm (bottom) throughout one entire cooling (black) and reheating cycle (red). Absorption profiles at 295 nm indicate that quadruplex formation is complete at approximately 35 °C. Decrease in thermal stability of the G-quadruplex is absent at pH 4.4 (blue). (B) Temperature-dependent CD spectra after subtraction of both duplex and buffer contribution. Positive bands appear around 260 and 295 nm, suggesting the onset of G-quadruplex formation at approximately 60 °C. (C) Temperature-dependent CD spectra after subtraction of the buffer contribution. The isoelliptic point in the relevant temperature range (40 °C - 10 °C, red circle) suggests that only the population of duplex and single strand change in response to alterations in temperature.

Supplementary Tables

Supplementary Table S1. UV melting data. Experimental error is given in ± 1σ. Propagation of experimental error: $\sigma(\Delta(x_1, x_2)) = \sqrt{\sigma_1^2 + \sigma_2^2}$

Sequence pair	pH	Exp. Tm (°C)	ΔTm (°C)	ΔH (kJ mol^{-1})	ΔS (J K^{-1} mol^{-1})	ΔG° (kJ mol^{-1})	ΔΔG° (kJ mol^{-1})
top1, bottom1		58.6 ± 0.7	-	-336.4 ± 11.5	-902.0 ± 32.9	-67.5 ± 1.7	-
top1::GQ1, bottom1		52.7 ± 2.5	5.9 ± 2.6	-252.2 ± 56.6	-585.0 ± 98.2	-52.7 ± 3.4	14.8 ± 3.8
top1::GQ2, bottom1		51.4 ± 1.8	7.2 ± 1.9	-213.8 ± 11.0	-547.0 ± 34.8	-50.7 ± 1.3	16.8 ± 2.2
top1::poly(T)1, bottom1		56.9 ± 0.7	1.7 ± 1.0	-332.8 ± 12.1	-896.7 ± 36.4	-65.5 ± 1.4	2.0 ± 2.2
top1::poly(T)2, bottom1		56.5 ± 0.5	2.1 ± 0.8	-333.0 ± 30.6	-898.5 ± 93.9	-65.1 ± 2.6	2.4 ± 3.1
top1::poly(T)3, bottom1		57.2 ± 0.5	1.3 ± 0.9	-336.1 ± 23.4	-905.4 ± 72.2	-66.1 ± 1.9	1.4 ± 2.6
top1, bottom1-1MM	7.4	56.9 ± 0.2	-	-344.7 ± 16.2	-932.6 ± 49.5	-65.9 ± 0.6	-
top1::GQ2, bottom1-1MM		50.9 ± 1.3	5.9 ± 1.4	-216.0 ± 4.0	-555.2 ± 15.1	-50.5 ± 0.6	15.4 ± 0.9
top1, bottom1-2MM		50.3 ± 0.4	-	-256.4 ± 17.6	-681.0 ± 54.3	-53.4 ± 1.4	-
top1::GQ2, bottom1-2MM		50.1 ± 1.0	0.2 ± 1.1	-202.1 ± 4.1	-514.6 ± 7.2	-48.7 ± 2.0	4.7 ± 2.4
top1, bottom1-3MM		50.6 ± 0.9	-	-218.6 ± 9.8	-563.3 ± 31.1	-50.6 ± 0.7	-
top1::GQ2, bottom1-3MM		50.8 ± 1.2	0 ± 1.5	-217.2 ± 57.3	-560.3 ± 176.8	-50.2 ± 4.8	0.4 ± 4.8

Supplementary Table S2. CD melting data.

Sequence pair	pH	Exp. Tm (°C)	ΔTm (°C)	ΔH (kJ mol^{-1})	ΔS (J K^{-1} mol^{-1})	ΔG° (kJ mol^{-1})	ΔΔG° (kJ mol^{-1})
top1, bottom1	7.4	57.3 ± 0.3	-	-327.2 ± 7.2	-891.7 ± 22.1	-61.4 ± 13.8	-
top1::GQ2, bottom1		62.4 ± 1.3	0 ± 1.3	-223.5 ± 0.8	-564.5 ± 2.4	-55.3 ± 1.6	7.3 ± 13.9
top1::poly(T)1, bottom1		61.1 ± 0.2	0 ± 0.3	-294.5 ± 5.4	-780.7 ± 16.2	-61.8 ± 10.2	0.8 ± 17.2
top1, bottom1-3MM		52.4 ±1.1	-	-219.2 ± 2.5	-571.6 ± 0.1	-48.8 ± 2.9	-
top1::GQ2, bottom1-3MM		57.5 ± 1.4	5.1 ± 1.7	-215.1 ± 0.8	-549.0 ± 0.7	-51.4 ± 1.0	0 ± 3.1

Supplementary Table S3. UV melting data. Experimental error is given in ± 1σ. Propagation of experimental error: $\sigma(\Delta(x_1, x_2)) = \sqrt{\sigma_1^2 + \sigma_2^2}$

Sequence pair	pH	Exp. Tm (°C)	ΔTm (°C)	ΔH (kJ mol^{-1})	ΔS (J K^{-1} mol^{-1})	ΔG° (kJ mol^{-1})	ΔΔG° (kJ mol^{-1})
top2, bottom2		54.3 ± 0.5	-	-381.2 ± 7.4	-1052.3 ± 23.5	-67.5 ± 0.6	-
top2::GQ2, bottom2		49.9 ± 0.3	4.4 ± 0.6	-245.7 ± 14.6	-648.8 ± 44.8	-52.3 ± 1.2	15.2 ± 1.4
top2, bottom2-1MM		56.4 ± 0.4	-	-352.2 ± 19.6	-956.8 ± 58.8	-66.9 ± 2.1	-
top2::GQ2, bottom2-1MM		50.6 ± 0.9	5.9 ± 1.0	-262.0 ± 14.9	-698.3 ± 45.3	-53.8 ± 1.5	13.1 ± 2.6
top2, bottom2-2MM		54.1 ± 0.6	-	-327.7 ± 19.8	-889.5 ± 59.4	-62.5 ± 2.1	-
top2::GQ2, bottom2-2MM		50.0 ± 1.0	4.0 ± 1.1	-235.3 ± 10.9	-616.3 ± 33.8	-51.5 ± 1.0	10.9 ± 2.4
top2, bottom2-3MM		52.3 ± 0.5	-	-287.9 ± 11.3	-772.9 ± 34.0	-57.5 ± 1.2	-
top2::GQ2, bottom2-3MM	7.4	48.4 ± 1.4	3.9 ± 1.5	-238.9 ± 9.6	-632.6 ± 26.2	-50.3 ± 2.0	7.2 ± 2.2
top2, bottom2-4MM		51.4 ± 1.1	-	-262.7 ± 11.1	-698.5 ± 34.7	-54.4 ± 0.8	-
top2::GQ2, bottom2-4MM		47.8 ± 1.0	3.5 ± 1.5	-226.1 ± 12.7	-593.4 ± 40.7	49.2 ± 1.1	5.1 ± 1.4
top2, bottom2-5MM		50.4 ± 0.8	-	-228.3 ± 13.8	-594.0 ± 43.6	-51.2 ± 0.9	-
top2::GQ2, bottom2-5MM		46.2 ± 2.2	4.3 ± 2.4	-214.7 ± 4.7	-558.2 ± 15.6	-48.2 ± 1.7	3.0 ± 1.9
top2, bottom2-6MM		49.9 ± 2.6	-	-206.6 ± 13.3	-527.5 ± 39.7	-49.3 ± 2.1	-
top2::GQ2, bottom2-6MM		50.8 ± 3.0	0 ± 4.0	-207.9 ± 19.4	-530.7 ± 58.9	-49.7 ± 2.6	0 ± 3.3

Supplementary Table S4. UV melting data. Experimental error is given in ± 1σ. Propagation of experimental error: $\sigma(\Delta(x_1, x_2)) = \sqrt{\sigma_1^2 + \sigma_2^2}$

Sequence pair	pH	Exp. Tm (°C)	ΔTm (°C)	ΔH (kJ mol^{-1})	ΔS (J K^{-1} mol^{-1})	ΔG° (kJ mol^{-1})	ΔΔG° (kJ mol^{-1})
top2, bottom2		39.4 ± 0.9	-	-254.9 ± 16.5	-703.9 ± 51.9	-45.1 ± 1.2	-
top2::IM, bottom2		35.2 ± 0.8	4.1 ± 1.2	-254.0 ± 2.1	-720.7 ± 4.3	-39.0 ± 1.0	6.0 ± 1.6
top2, bottom2-1MM		39.9 ± 1.0	-	-248.9 ± 9.3	-683.4 ± 28.7	-45.2 ± 1.1	-
top2::IM, bottom2-1MM		35.3 ± 0.7	4.6 ± 1.3	-218.0 ± 14.0	-596.5 ± 37.5	-40.9 ± 0.5	4.3 ± 1.2
top2, bottom2-2MM	4.0	35.0 ± 1.9	-	-236.3 ± 1.5	-657.9 ± 2.1	-40.2 ± 0.9	-
top2::IM, bottom2-2MM		33.2 ± 1.8	1.8 ± 2.6	-218.0 ± 5.9	-607.4 ± 20.3	-36.9 ± 0.5	3.3 ± 1.0
top2, bottom2-3MM		31.8 ± 0.8	-	-188.4 ± 1.9	-506.5 ± 6.4	-37.3 ± 0.1	-
top2::IM, bottom2-3MM		29.7 ± 0.9	2.1 ± 1.2	-191.3 ± 3.3	-515.7 ± 8.7	-37.5 ± 0.7	0 ± 0.7

Supplementary Table S5. UV melting data. Experimental error is given in ± 1σ. Propagation of experimental error: $\sigma(\Delta(x_1, x_2)) = \sqrt{\sigma_1^2 + \sigma_2^2}$

Sequence pair	pH	Exp. Tm (°C)	ΔTm (°C)	ΔH (kJ mol^{-1})	ΔS (J K^{-1} mol^{-1})	ΔG° (kJ mol^{-1})	ΔΔG° (kJ mol^{-1})
top2, bottom2	4.0	39.4 ± 0.9	-	-254.9 ± 16.5	-703.9 ± 51.9	-45.1 ± 1.2	-
top2::GQ2, bottom2	4.0	27.8 ± 0.4	11.6 ± 1.0	-233.3 ± 8.2	-663.5 ± 26.2	-35.5 ± 0.4	9.6 ± 1.3
top2::IM, bottom2	4.0	35.2 ± 0.8	4.1 ± 1.2	-254.0 ± 2.1	-720.7 ± 4.3	-39.1 ± 1.0	6.0 ± 1.6
top2, bottom2	4.4	43.6 ± 1.5	-	-337.6 ± 19.8	-953.9 ± 65.6	-53.2 ± 1.1	-
top2::GQ2, bottom2	4.4	34.7 ± 1.1	8.9 ± 1.8	-224.6 ± 7.0	-617.7 ± 22.9	-40.4 ± 0.8	12.8 ± 1.4
top2::IM, bottom2	4.4	40.3 ± 0.7	3.4 ± 1.6	-326.6 ± 22.4	-929.7 ± 69.2	-49.4 ± 1.9	3.8 ± 2.2
top2::IM, GQ2::bottom2	4.4	41.1 ± 1.4	2.6 ± 2.0	-324.5 ± 21.0	-920.9 ± 65.7	-49.9 ± 1.9	3.3 ± 2.3
top2::GQ2, IM::bottom2	4.4	40.9 ± 2.1	2.1 ± 2.8	-206.1 ± 12.3	-545.0 ± 43.3	-43.6 ± 0.7	9.6 ± 0.7
top1, bottom2	5.0	52.0 ± 0.4	-	-360.1 ± 4.1	-995.2 ± 12.8	-63.4 ± 0.8	-
top2::GQ2, bottom2	5.0	43.9 ± 1.0	8.0 ± 1.1	-234.9 ± 9.0	-629.2 ± 27.7	-47.3 ± 1.0	16.1 ± 1.3
top2, bottom2	5.4	53.7 ± 0.3	-	-369.7 ± 6.8	-1018.9 ± 19.5	-66.0 ± 1.1	-
top2::GQ2, bottom2	5.4	46.0 ± 0.6	7.7 ± 0.6	-236.7 ± 11.5	-631.5 ± 38.3	-48.4 ± 1.0	17.5 ± 1.5
top2, bottom2	5.9	54.9 ± 0.4	-	-363.9 ± 6.4	-997.7 ± 18.6	-66.5 ± 0.9	-
top2::GQ2, bottom2	5.9	48.2 ± 0.6	6.6 ± 0.7	-244.2 ± 10.5	-648.0 ± 32.2	-51.0 ± 0.9	15.5 ± 1.3
top2, bottom2	7.40	54.3 ± 0.5	-	-381.2 ± 7.4	-1052.3 ± 23.5	-67.5 ± 0.6	-
top2::GQ2, bottom2	7.40	49.9 ± 0.3	4.4 ± 0.6	-245.7 ± 14.6	-648.8 ± 44.8	-52.3 ± 1.2	15.2 ± 1.4

Supplementary References

1. Leroy, J.-L., Guéron, M., Mergny, J.L. and Hélène, C. (1994) Intramolecular folding of a fragment of the cytosine-rich strand of telomeric DNA into an i-motif. *Nucleic Acids Res.*, **22**, 1600-1606.
2. Mergny, J.L., Phan, A.T. and Lacroix, L. (1998) Following G-quartet formation by UV-spectroscopy. *FEBS Lett.*, **435**, 74-78.
3. Li, X., Peng, Y., Ren, J. and Qu, X. (2006) Carboxyl modified single-walled carbon nanotubes selectively induce human telomeric i-motif formation. *Proc. Natl. Acad. Sci. USA*, **103**, 19658-19663.
4. Kendrick, S., Akiyama, Y., Hecht, S.M. and Hurley, L.H. (2009) The i-motif in the bcl-2 P1 promoter forms an unexpectedly stable structure with a unique 8:5:7 loop folding pattern. *J. Am. Chem. Soc.*, **131**, 17667-17676.
5. Mergny, J.L. and Lacroix, L. (2003) Analysis of thermal melting curves. *Oligonucleotides*, **13**, 515-537.
6. Turner, D.H. (2000) In Bloomfield, V. A., Crothers, D. M. and Tinoco, I. J. (eds.), *Nucleic acids: structures, properties, and functions*. University Science Books, Sausalito, SA 94965.
7. Smith, J.D., Cappa, C.D., Wilson, K.R., Cohen, R.C., Geissler, P.L. and Saykally, R.J. (2005) Unified description of temperature-dependent hydrogen-bond rearrangements in liquid water. *Proc. Natl. Acad. Sci. USA*, **102**, 14171-14174.
8. Damerla, R.R., Knickelbein, K.E., Kepchia, D., Jackson, A., Armitage, B.A., Eckert, K.A. and Opresko, P.L. (2010) Telomeric repeat mutagenicity in human somatic cells is modulated by repeat orientation and G-quadruplex stability. *DNA Repair*, **9**, 1119-1129.
9. Arora, A., Nair, D.R. and Maiti, S. (2009) Effect of flanking bases on quadruplex stability and Watson-Crick duplex competition. *FEBS J.*, **276**, 3628-3640.
10. Vorlíčková, M., Kejnovská, I., Sagi, J., Renčiuk, D., Bednářová, K., Motlová, J. and Kypr, J. (2012) Circular dichroism and guanine quadruplexes. *Methods*, **57**, 64-75.
11. Chen, Y.-H., Yang, J.T. and Martinez, H.M. (1972) Determination of the secondary structures of proteins by circular dichroism and optical rotatory dispersion. *Biochemistry*, **11**, 4120-4131.
12. Bucek, P., Jaumot, J., Aviñó, A., Eritja, R. and Gargallo, R. (2009) pH-modulated Watson-Crick duplex-quadruplex equilibria of guanine-rich and cytosine-rich DNA sequences 140 base pairs upstream of the c-kit transcription initiation site. *Chem. Eur. J.*, **15**, 12663-12671.
13. Dominguez-Martín, A., Johannsen, S., Sigel, A., Operschall, B.P., Song, B., Sigel, H., Okruszek, A., González-Pérez, J.M., Gutiérrez-Niclós, J. and Sigel, R.K.O. Intrinsic acid-base properties of a hexa-2'-deoxynulceoside pentaphosphate, d(ApGpGpCpCpT). Neighboring effects and isomeric equilibria. *Chem. Eur. J.*, in press.
14. Datta, K., Johnson, N.P. and von Hippel, P.H. (2010) DNA conformational changes at the primer-template junction regulate the fidelity of replication by DNA polymerase. *Proc. Natl. Acad. Sci. USA*, **107**, 17980-17985.
